# The impact of a brief mindfulness training on interoception: A randomized controlled trial

**DOI:** 10.1371/journal.pone.0273864

**Published:** 2022-09-07

**Authors:** Geissy Lainny de Lima-Araujo, Geovan Menezes de Sousa Júnior, Thatiane Mendes, Marcelo Demarzo, Norman Farb, Draulio Barros de Araujo, Maria Bernardete Cordeiro de Sousa

**Affiliations:** 1 Brain Institute, Federal University of Rio Grande do Norte, Natal, Brazil; 2 Department of Psychology, University of Toronto Mississauga, Mississauga, Canada; 3 Mente Aberta—Brazilian Center for Mindfulness and Health Promotion–Universidade Federal de São Paulo (UNIFESP), São Paulo, Brazil; Brown University, UNITED STATES

## Abstract

Interoception is a collection of different representations of signals originating within the body. The way of perceiving these signals seems to be related to both emotion regulation and dysregulation, and its dysfunction is implicated across a variety of affective disorders. There is a growing body of research investigating the relationship between mindfulness meditation practices and interoception showing an increase in interoceptive processes with regular training. In this study, we assessed the effects of a three-day mindfulness training on interoceptive accuracy and sensibility in a young healthy adult sample. Moreover, we also performed a mediation analysis on interoceptive sensibility and anxiety. Healthy participants (n = 40) naive to mindfulness practices were randomized to a brief mindfulness training (MT) (n = 20, females = 10) or to an active control group (n = 20, females = 10). Participants were assessed before and after the 3-days intervention for both groups on measures of interoception and anxiety in a modified intention-to-treat approach. The brief mindfulness training group increased interoceptive sensibility while active control had no effects on this variable. Five out of eight subdomains of interoceptive sensibility were significantly improved after mindfulness training. There was no significant difference in interoceptive accuracy after training. The effect of a brief mindfulness training on interoceptive sensibility mediated changes in the anxiety state. To date, this is the first study showing a plausible mechanism of a brief mindfulness training to explain the anxiolytic effects of meditation practices.

**Trial registration:** RBR-7b8yh8, March 28^th^ 2017 http://www.ensaiosclinicos.gov.br/rg/RBR-7b8yh8/.

## Introduction

Interoception is an interactive process of receiving, accessing, appraising, and integrating inner bodily signals, and it depends on past experiences and current individual mindset [1]. The growing interest in understanding interoception is due to its relationship to emotions and feelings in healthy and clinical populations [2–5]. Over the last decade, there has been a variety of evidence linking interoceptive dysfunction to depression, anxiety disorders, eating disorders, chronic pain, and others [5–8]. Interoceptive signals are not thought to be intrinsically wholesome or harmful; rather, how one attends to interoceptive signals may differentiate between adaptive and maladaptive responses to interoceptive cues [9]. For example, anxiety may stem from the conditioned appraisal of body sensations as threat signals, promoting emotional fragility when compared to a more mindful or decentered attentional orientation [10].

Embodying a mindful attitude towards feelings, sensations, and thoughts especially based on acting with acceptance, seems to lead to important changes in coping and emotion regulation strategies [11]. One way of training this sort of attentional style is through mindfulness practices that aim to cultivate an open, non-judgmental, curious way of dealing with the experience in the present moment [12, 13]. Over the last few decades, mindfulness-based exercises have been implemented in clinical and non-clinical settings in programs and interventions of varying duration [12, 14–16]. Mindfulness training (MT) has been associated with significant improvements in cognition and affect, including improvement of attentional skills and reduction of stress markers, depressive symptoms, and anxiety-like behaviors [17–20].

Brief mindfulness training is gaining attention due to its potential to improve well-being even after just a few days of practice [21–24]. Mindfulness-based training contains meditation exercises that can be conceptualized as "a family of complex emotional and attentional regulatory training regimes developed for various ends, including the cultivation of well-being and emotional balance” [25]. There are some strategies or styles used to apply the meditation methods as mental training such as focused attention and open monitoring that can result in increased states of consciousness that facilitate wellbeing [25]. In this study, we consider mindfulness as mental training using focused attention exercises (body scan and breath awareness). Moreover, we follow the type of nomenclature of mindfulness-based activities of Heppner and Shirk that points out the characterization of brief mindfulness-based training as: i) The employment of multiple mindfulness exercises or sessions, over a relatively short period of time. ii) The individual sessions are similar to those in mindfulness inductions but usually involve multiple days (and usually no longer than a week). iii) Utilized to promote a longer and more comprehensive mindfulness state and typically implemented in non‐clinical samples.” [26].

In a recent publication of our group, we showed that a 3-days mindfulness-based intervention along with an evaluation of mindfulness trait leads to an important modulation of psychophysiological measures of well-being such as perceived stress, cortisol, anxiety state, positive and negative affect [23]. In this previous study, we observed through a moderated mediation analysis, that a brief meditation training increases positive affect and decreases perceived stress. In addition, a partial mediation effect of the mindfulness state acts by decreasing the state of anxiety [23]. We also observed a decrease in anxiety state after mindfulness training (S2 Fig).

One possible theory for MT’s effects on well-being is the improvement of the relationship with interoceptive cues by promoting a more adaptative behavior towards body sensations with less experiential avoidance [27–30]. In fact, interoception seems to be modulated by attentional training with contemplative practices, although recent evidence suggest that meditation do not have significant effect on interoceptive accuracy [31, 32]. Interoception has different representations, including accuracy which corresponds to performance in objective signal detection tasks, and sensibility, characterized as a qualitative report of body sensation [30]. Most attempts to assess individual differences in interoceptive accuracy have used measures of the cardiovascular system, primarily because heartbeats counting tasks allow internal body information to be measured easily and objectively [33]. People with high interoceptive accuracy can experience emotions more intensely, although there is no difference in arousal represented by sympathetic activation between good and poor heart rate detectors [34]. To assess interoceptive sensibility is necessary to investigate the personal experience of internal body sensation through self-assessed measures [35]. This psychological dimension related to the perception and interpretation of the intensity of the experience is an important key factor to explain the relationship between interoceptive ability in anxious individuals [10, 36].

The current research is guided by contemporary psychological and contemplative theory on mental health promotion. From this perspective, interoception is thought to provide the ‘raw material’ for emotional appraisal, which at a minimum involves interpreting physiological change for its survival relevance. In this way, interoception informs our momentary assessment of coping capacity and motivation to act, as well as broader appraisals of well-being. Over time, many of these appraisals are thought to become so highly rehearsed as to appear automatic and obligatory, with powerful implications for well-being. A given interoceptive signal such as elevated heartbeat may indicate excitement and enthusiasm to one person, but prophesize catastrophe and loss of control to another. Mindfulness training emphasizes granular, nonjudgmental attention to momentary experience. When practiced successfully, MT inhibits the conditioned association between sensory signals and cognitive appraisals [1].

Some studies involving anxiety disorders show that some individuals increased self-report of somatic sensations and also present a dysfunctional cognitive appraisal of these sensations with a significant bias toward a catastrophizing and danger-related interpretational style especially arising from the cardiac systems is found [36]. Although this possibly explains why a mindful attitude toward body sensations is key to establishing better emotion regulation strategies [11], the mechanisms underlying the efficacy of brief mindfulness practices are not clearly understood, limiting opportunities to refine and extend mindfulness for promoting well-being. To address this gap in the literature, we designed this study to assess the effects of a three-day mindfulness training on interoceptive accuracy and sensibility in a young healthy adult sample. Moreover, we also evaluated a potential mediation role of interoception sensibility on anxiety. We tested the following hypotheses: 1) A brief mindfulness-based training improves different domains of interoception and 2) Improvement in interoception mediates lower levels of anxiety after mindfulness training.

## Materials and methods

This study is a parallel-arm randomized clinical trial approved by the ethics committee of the Federal University of Rio Grande do Norte (UFRN/Brazil, #55193416.4.0000.5537) and registered in the Brazilian Clinical Trials platform (RBR-7b8yh8). Data collection occurred between April 2017-June 2018 in Natal/Brazil. The research was advertised on campus and by an online platform where participants could apply as potential candidates for the study about attentional practices by filling up a survey containing questions about sociodemographic information, health, and yoga/meditation practice. Written informed consent was obtained from all selected participants prior to the study, and they did not receive any financial reward or course credits for participating. All methods and experiments were performed in accordance with ethical guidelines and relevant regulations. The complete study protocol can be found in the attached trial protocol document.

### Participants

**Fig 1:**
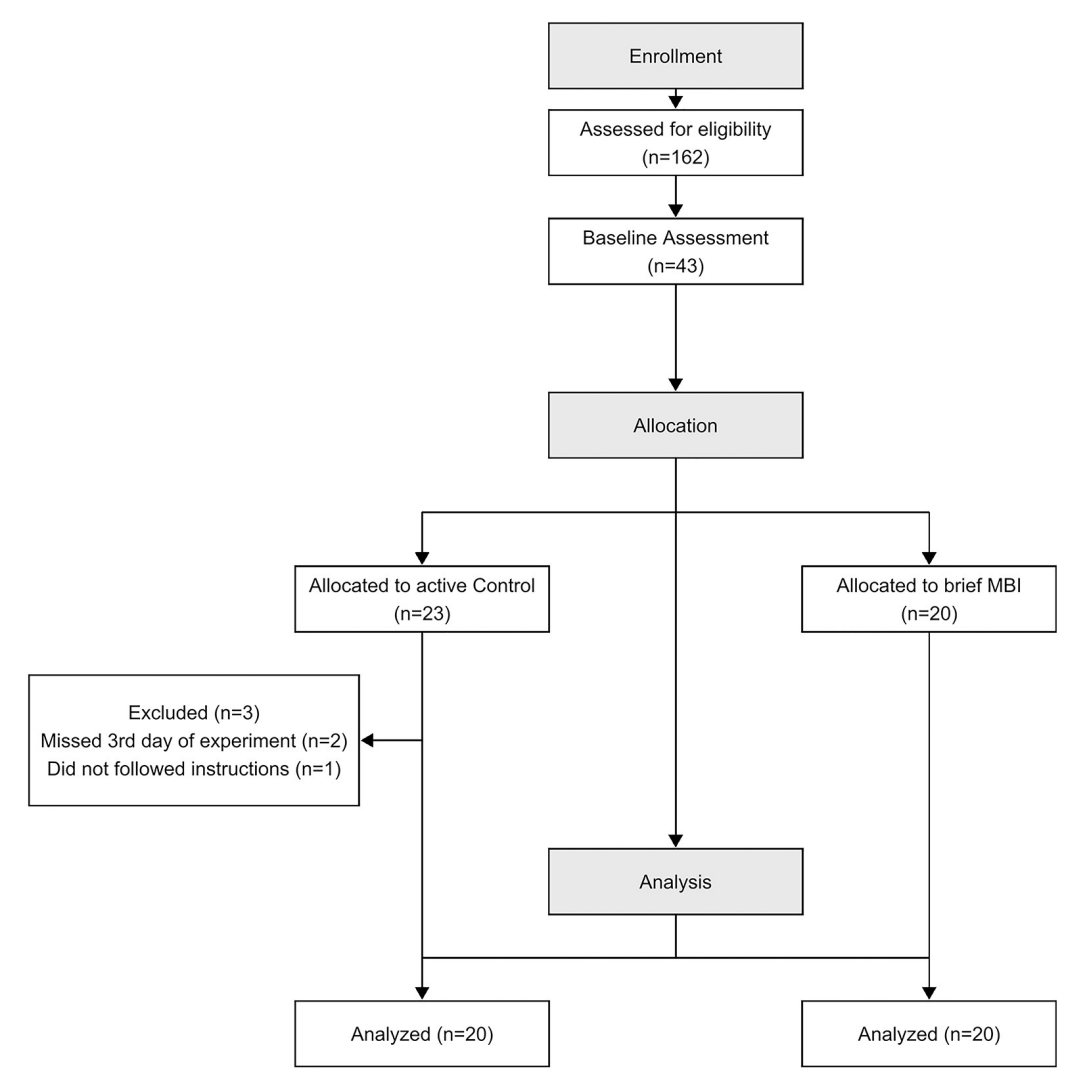
CONSORT flow diagram of the study.

Forty-three healthy graduate and undergraduate students with no previous meditation or yoga practice were eligible to participate in the study after a baseline assessment via an online sign-up platform (Fig 1).

All participants were right-handed, with normal to corrected-to-normal vision, and no previous metabolic or psychiatric disorders. Participants were not using psychotropic or anti-inflammatory medications, as self-declared at the initial assessment. Participants were age and sex-matched to take part in the study in the same week. In other words, we always had two males or two females of the same age coming to the lab for 3 days of the same week. When they arrived at the laboratory, the first participant had access to a box where they blindly chose one of the paper ballots containing one of the two groups in which they would be part. The next participant was allocated to the other group and this procedure was performed in this sequence until finalizing the distribution of participants in the desired sample. The sample size calculation is described in our previous publication [23].

Three participants were excluded from the study. Two of them missed the third day of the experiment, and one did not follow the instructions for the last day’s tasks. Data from the remaining forty participants–aged between 18 to 30 (mean = 24.15; SD = 3.56), in mindfulness training (MT group, n = 20, females = 10, mean ± SD age: 24.05 ± 3.76) or active control group (AC, n = 20, females = 10, mean ± SD age: 24.25 ± 3.55) were analyzed. In other words, we included in the analysis all volunteers who attended the baseline and session 2. Participants had similar sociodemographic characteristics, and baseline measurements between groups were not significant (Table 1).

**Table 1:** Sociodemographic characteristics of the sample and baseline comparisons of scales between groups.

		Control	Mindfulness	χ^2^/W	p-value
**Sociodemographic characteristics**					
Participants		20	20	0	1.00
Sex	Male:Female	10:10	10:10	0	1.00
Age	Mean (SD)	24.25 (3.55)	24.05 (3.76)	201	0.99
Ethnicity	White	6	9	1.13	0.77
	Pardo	11	9		
	Black	1	1		
	Not declared	2	1		
Scholarity	13–15 years	10	10	0	1.00
	16–19 years	4	4		
	Up to 20 years	6	6		
Civil state	Single	18	19	0	1.00
	Married	2	1		
**Baseline comparisons**					
MAIA	Mean (SD)	21.4 (5.41)	20.7 (3.41)	232	0.40
Noticing	Mean (SD)	3.69 (0.95)	3.36 (0.81)	249	0.19
Not-Distracting	Mean (SD)	1.58 (0.84)	1.80 (0.76)	182	0.63
Not-Worrying	Mean (SD)	2.20 (1.18)	2.43 (0.95)	183.5	0.66
Attention Regulation	Mean (SD)	1.91 (1.13)	1.74 (0.67)	222	0.56
Emotional Awareness	Mean (SD)	3.47 (1.36)	3.32 (1.15)	227	0.47
Self-Regulation	Mean (SD)	2.62 (1.03)	2.35 (0.94)	235.5	0.34
Body Listening	Mean (SD)	2.58 (0.89)	2.43 (0.67)	238.5	0.30
Trusting	Mean (SD)	3.35 (1.01)	3.27 (1.01)	207	0.86
State Anxiety	Mean (SD)	38.6 (9.43)	39.7 (7.03)	144	0.13
Interoceptive Accuracy	Mean (SD)	0.54 (0.32)	0.54 (0.26)	202	0.97

χ^2^ = Chi-squared association test statistic; W = Wilcoxon sum-rank test statistic; SD = standard deviation.

### Inclusion and exclusion criteria

Eligibility requirements include age between 18 and 30 years, no previous regular formal experience with yoga or meditation practice; no self-declared psychiatric or metabolic disorders; not taking any psychotropic medication at the time of the study; right-handed. Exclusion criteria include situations of non-attendance on all days of the study; presenting any signs and/or symptoms of infectious disease at the time of the study; not following or understanding task instructions.

### Training sessions

The training sessions had the same duration for both groups (30 minutes a day for three consecutive days). Both interventions were delivered in person, individually, in the university’s research laboratory for 3 consecutive days where participants listened to previously recorded audio with headphones.

Control group participants listened to 3 different audios on educational health for about 15 minutes, answered one question about the audio content, and then colored the selected pictures for time matching the mindfulness training session. The content aimed to inform and educate about scientific discovery and its application in daily life. All the transcripts, audios, and figures used in this study can be found in the open science framework (link available at the data availability session).

Participants in the mindfulness group underwent a 30-minute mindfulness-based exercise containing a standard mindfulness meditation (body scan and breath awareness) with the same audio for all the 3 sessions. Instructions included acquiring a sitting comfort position, eyes closed, and maintaining a natural breathing rhythm establishing a mindful attitude towards body and breath sensations over time. Mindfulness practice was recorded by a qualified instructor trained by the Brazilian Center of Mindfulness in a 1-year teaching training program, including experience in facilitating groups, personal practice, and supervision. As researchers, we presented the study and explained to the participants about attentional training and task procedures so that they could not easily differentiate the groups. Before starting the training, the same instructions were given to all participants, in the same way, that is, listening to the audio and being attentive to all the tasks during the 30 minutes of training.

On the first day of the intervention, baseline data were collected by applying questionnaires and measuring neurophysiological data. After that, participants performed the training session. On the second day, participants were only submitted to the training session. Finally, on the third day, participants performed the training, and then questionnaires and neurophysiological data were collected.

### Assessments

All participants went through two evaluation phases, one on the first day, immediately before training, after the randomization protocol, and another on the third day immediately after the training session. Assessments included psychophysiological measures of well-being as reported in our previous publication [23], electroencephalography, and interoception. In this study, we focus on measures of interoceptive sensibility, interoceptive accuracy, and the relationship between these measures with levels of state anxiety. All the questionnaires used in this study were previously translated and used in the Brazilian population and the reference for such instruments are cited along with the text.

- **Interoceptive accuracy.** Interoceptive accuracy (IAc) was measured using the heartbeat counting task [33]. This task consists of silently counting heartbeats during different periods (e.g., 25s, 35s, 45s) without manual or tactual feedback. At the same time, to objectively measure cardiac signals, heartbeats were continuously recorded with passive Ag/AgCL electrodes placed on Lead II configuration connected to BrainAmp EXG amplifiers and registered with BrainVision Recorder software version 1.20.0506 (Brain Products, GmbH). In this study, all the participants were evaluated in the supine position and instructed to focus attention on their heartbeat during the period between two soft tones presented at the beginning and the end of the task. Every participant went through a training session where they received the instructions and performed a dry run to familiarize themselves with the task. The test was performed three times with three distinct length periods: 25s, 35s, and 45s, with 9 trials interspersed with 30s intervals. After the stop tone, participants were asked to report the number of heartbeats mentally counted. Importantly, they did not receive any prior information about the length intervals or how many heartbeats occurred after the count time. All trials were pseudorandomized. To calculate the IAc, we applied the following formula [33, 37].
$$IAc=\frac{1}{9}\sum \frac{1-|recordedheartbeats-countedheartbeats|}{recorded\;heartbeats}$$
$$IAc=\frac{1}{9}\sum \frac{1-|Hb_{r}-Hb_{c}|}{Hb_{r}}$$
  Where *Hb_r_* and *Hb_c_* stands for recorded and counted heartbeats, respectively. The resulting formula score ranges from 0 to 1, with higher scores indicating better interoceptive accuracy, so a maximum score of 1 indicates perfect accuracy of heartbeat perception.
- **Interoceptive sensibility.** The Multidimensional Assessment of Interoceptive Awareness (MAIA) [35] aims to assess the ability to perceive emotions and bodily sensations and is characterized as a measure of interoceptive sensibility. It is a 32-item scale with eight subscales related to the emotional reaction to bodily sensations, awareness of body sensations, emotional regulation, body-mind awareness, and trust in body sensations. The eight subscales are named: “*Noticing”*, *“Not-distracting”*, *“Not-worrying”*, *“Attention Regulation”*, *“Emotional Awareness”*, *“Self-regulation”*, *“Body Listening” and “Trusting”*. Internal consistency (Cronbach’s alpha, α) for this instrument was good for our sample (MAIA, α = 0.89). The Portuguese version of MAIA presents good psychometric properties for healthy adults [38].
- **Anxiety state.** State anxiety was measured by the 20-item self-reported State domain of the State-Trait Anxiety Inventory (STAI-S), which is scored on a four-point Likert scale [39]. State anxiety can be defined as a transitory and momentary emotional state that results from situational stress. Internal consistency (Cronbach’s alpha, α) for this instrument was good for our sample (α = 0.90). The Brazilian Portuguese version of this instrument was used [40].

## Data analysis

To test the hypothesis that mindfulness training would lead to increased interoceptive measures, we performed a modified intent-to-treat analysis, including all volunteers who attended the baseline and session 2. Statistical analysis was performed using R software (3.6.0) and the RStudio interface (1.2.1335). Not all variables achieved normal distribution. For this reason, we decided to proceed with a non-parametric analysis applying the Mann-Whitney test of change scores (Post-Intervention (day 3)–Baseline (day 1)) to assess group differences over time. The Wilcoxon-signed rank test was performed to assess intragroup time-related changes (Baseline vs. Post-Intervention). We provide effect sizes (*r*) between- and within-groups in each figure. The effect size *r* was calculated using the *rcompanion* package and is classified as small, medium, and large when the estimate is around *r* ≤ 0.10, 0.10< *r* ≤ 0.30, and 0.30 < *r* ≤ 0.50, respectively [41]. The correlation between interoceptive accuracy and interoceptive sensibility was assessed by the Spearman correlation test. Moreover, based in our previous study which showed that a brief mindfulness training reduces anxiety state levels [23], we also predicted that changes in interoceptive domains would negatively correlate with levels of anxiety after mindfulness training in the same sample. As a reanalysis of this data, we performed a mediation analysis to assess whether the effect of an independent variable over a dependent one is caused by a third, the so-called mediator, using the nonparametric Bootstrap Confidence Intervals approach as the mediation method. Here, we use group (Control = 0, Mindfulness = 1) as the independent variable, changes in the score of anxiety measures as the dependent variable, and changes in the interoceptive domains (MAIA score or IAc index) as the mediator. Notably, only variables that significantly changed after the training were included in the mediation analysis. The analysis was performed using the package *processr* with bootstrap method (10,000 times). To determine statistical significance, we considered *p≤ 0.05 (two-tailed).

## Results and discussion

### A brief mindfulness-based training enhances interoceptive sensibility but does not affect interoceptive accuracy

**Fig 2:**
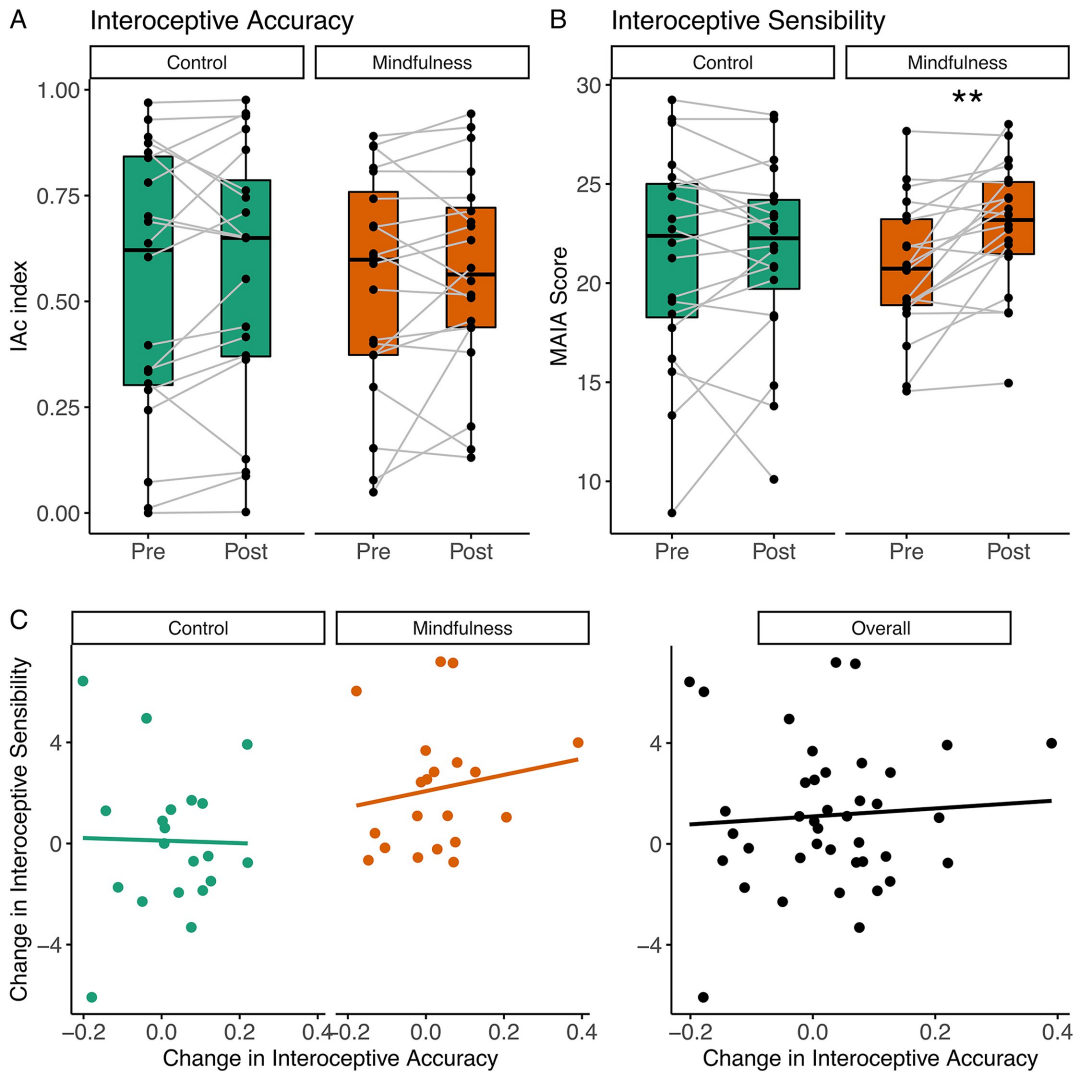
Interoceptive measures before and after training. **A)** Box-plots before and after intervention with measures of Interoceptive Accuracy (IAc) Index. Wilcoxon-signed rank test showed no significant intragroup difference for the Mindfulness group or the Control group. There was no between-group difference when comparing change scores (Post-Pre). **B)** Box-plots of Interoceptive Sensibility (Multidimensional Assessment of Interoceptive Awareness [MAIA] score) before and after intervention with values from all participants with connected dots. The Wilcoxon signed-rank test shows a significant within-group difference in interoceptive sensibility for the mindfulness group whereas there is no significant difference for the control group. **C)** Group comparison of scatterplots for interoception accuracy versus sensibility. These constructs were not correlated when analyzed separately by group and considering the scores of both groups together (D).

To test our first hypothesis and verify the impact of mindfulness training on domains of interoception, we evaluated measures of interoception accuracy and sensibility before and after completing a 3-days training. No training effects were observed on overall interoceptive accuracy (IAc; Fig 2A). Analysis of the change scores showed no significant difference between groups (p = 0.678, r = -0.07). Moreover, there was no differences within-group (Mindfulness group: p = 0.349, r = 0.22; Control group: p = 0.294, r = 0.24) for IAc. Additionally, we also performed a block analysis at the IAc task according to the length of the counting period (S1 Fig). No significant difference was found within or between groups, and details are provided in the supplemental material.

For interoceptive sensibility, a significant difference between groups was observed when comparing change scores for the MAIA scale (p = 0.020, r = 0.37). Moreover, we observed significantly higher scores after mindfulness training in a within-group analysis (p = 0.001, r = 0.68) (Fig 2B).

Interoceptive accuracy and sensibility were not correlated considering both baseline data from the two groups together (r_s_ = -0.004, p = 0.98), as well as within the mindfulness group (r_s_ = 0.21, p = 0.35) or the control group (r_s_ = -0.02, p = 0.91) (Fig 2C).

**Fig 3:**
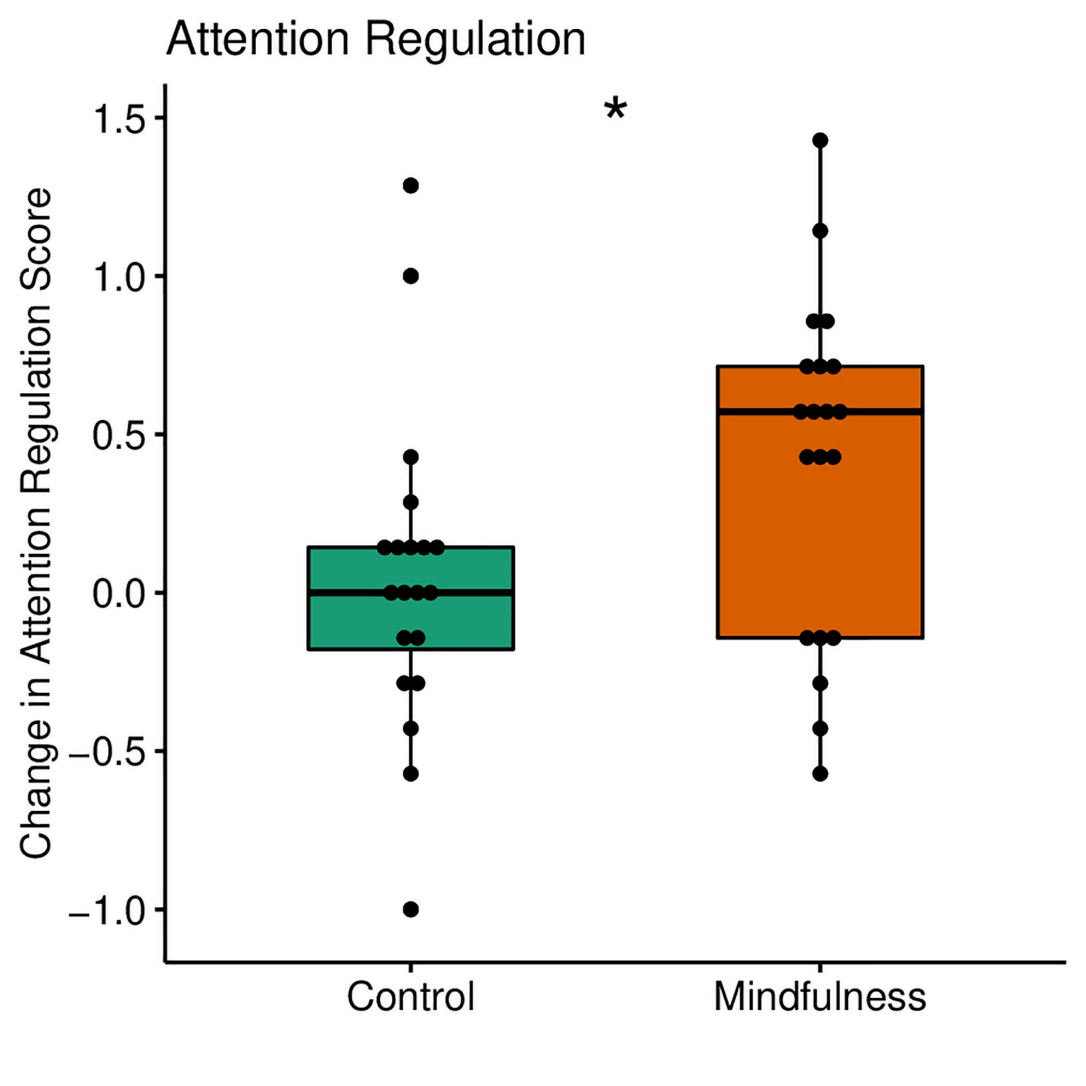
Box plots before and after intervention with change values of the attention regulation domain from the MAIA scale. The Mann-Whitney test showed a significant between-group difference for the attentional regulation domain, with an effect size from medium to high (r = 0.64).

We decided to investigate whether subdomains of the MAIA contributed differently to the change in the global score. When comparing groups by the change score (day 3—day 1), we found a significant difference only in the attention regulation subscale (p = 0.033, r = 0.34) (Fig 3).

**Fig 4:**
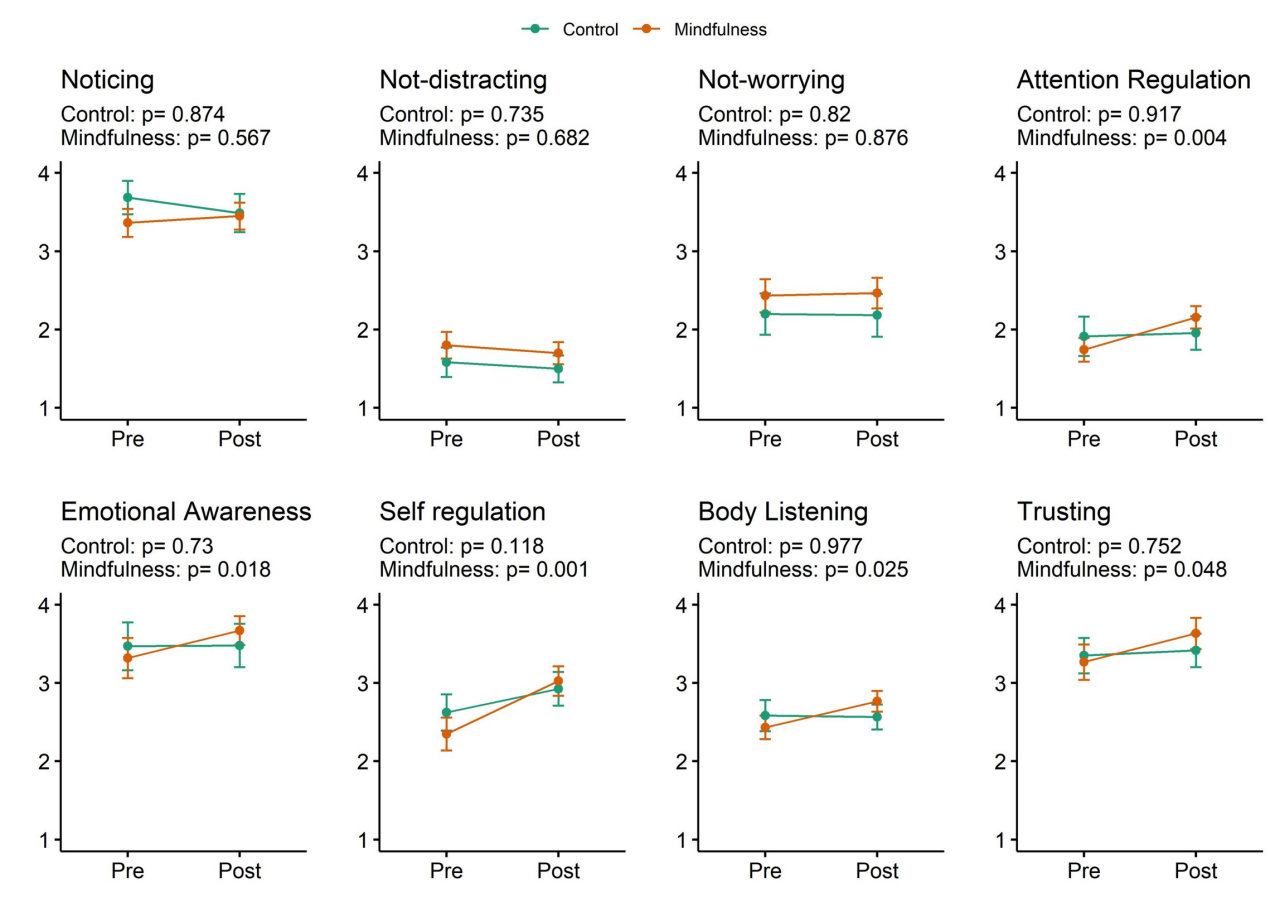
Total Multidimensional Assessment of Interoceptive Awareness (MAIA) domains score segmented by group and session. Lines represent the Mindfulness group (orange) and the Control group (green) before and after intervention with all MAIA subdomains. In 5 out of 8 MAIA domains, a significant increase was found in the Mindfulness but not in the Control group, as assessed by the Wilcoxon-signed rank test.

When compared within-group, we observed an impact of the mindfulness training among 5 out of the 8 subscales (Trusting: p = 0.048, r = 0.45; Self-Regulation: p = 0.001, r = 0.75; Attention Regulation: p = 0.004, r = 0.64; Body Listening, p = 0.025, r = 0.51; Emotional Awareness: p = 0.018, r = 0.54, representing 62% of the overall domain. In the active control group, there were no significant differences between sessions (Fig 4). Table 2 contains a summary of the measures presented in this study.

**Table 2:** Within and between groups comparisons for the control and mindfulness groups. Bold values denote statistical significance.

	Control				Mindfulness						
	*Within*				*Within*				Between		
	*Δ* (SD)	V	p-value	*r* [CI]	*Δ* (SD)	V	p-value	*r* [CI]	W	p-value	*r* [CI]
MAIA	0.10(2.88)	99	0.888	-.04 [-.48, .40]	2.16(2.50)	**23**	**0.001**	**.68 [.38, .85]**	**286**	**0.020**	**.37 [.08, .64]**
Noticing	-0.2(1.51)	55.5	0.874	-.04 [-.50, .39]	0.08(0.50)	43	0.567	.14 [-.35, .52]	216	0.669	.07 [-.24, .37]
Not-Distracting	-0.08(0.62)	75	0.735	-.08 [-.51, .37]	-0.1(0.69)	59.5	0.682	-.10 [-.50, .34]	203	0.945	.01 [-.29, .32]
Not-Worrying	-0.01(1.26)	55.5	0.820	.06 [-.35, .56]	0.03(0.80)	64.5	0.876	.04 [-.38, .48]	195	0.902	-.02 [-.32, .28]
Attention Regulation	0.04(0.49)	65.5	0.917	.03 [-.42, .51]	0.41(0.53)	**28**	**0.004**	**.64 [.32, .85]**	**279**	**0.033**	**.34 [.02, .63]**
Emotional Awareness	0.01(1.00)	46.5	0.730	.04 [-.37, .49]	0.35(0.56)	**26**	**0.018**	**.54 [.16, .77]**	237	0.320	.16 [-.18, .45]
Self-Regulation	0.3(0.83)	49.5	0.118	.35 [-.05, .70]	0.65(0.72)	**9**	**0.001**	**.75 [.54, .88]**	241	0.269	.18 [-.15, .46]
Body Listening	-0.01(0.8)	59	0.977	.01 [-.40, .51]	0.33(0.64)	**10**	**0.025**	**.51 [.17, .72]**	237.5	0.308	.16 [-.16, .46]
Trusting	0.06(0.72)	40.5	0.752	.08 [-.38, .52]	0.37(0.74)	**29.5**	**0.048**	**.45 [.03, .72]**	239	0.291	.17 [-.14, .46]
Anxiety State	0.00(5.65)	115	0.722	-.08 [-.53, .36]	-4.50(7.83)	**148**	**0.034**	**-.48 [-.74, -.09]**	148	0.162	-.22 [-.49, .07]
IAc	0.02(0.05)	76	0.294	.24 [-.23, .64]	0.01(0.04)	79	0.349	.22 [-.25, .64]	184	0.678	-.07 [-.39, .24]

*Δ* = post-intervention score–baseline; V = Wilcoxon signed-rank test statistic; *r* = effect size; CI = 95% confidence interval; W = Wilcoxon sum-rank test statistic; MAIA = Multidimensional Assessment of Interoceptive Awareness; IAc = Interoceptive accuracy index.

### Changes in interoceptive sensibility mediate the decrease in anxiety state after mindfulness training

**Fig 5:**
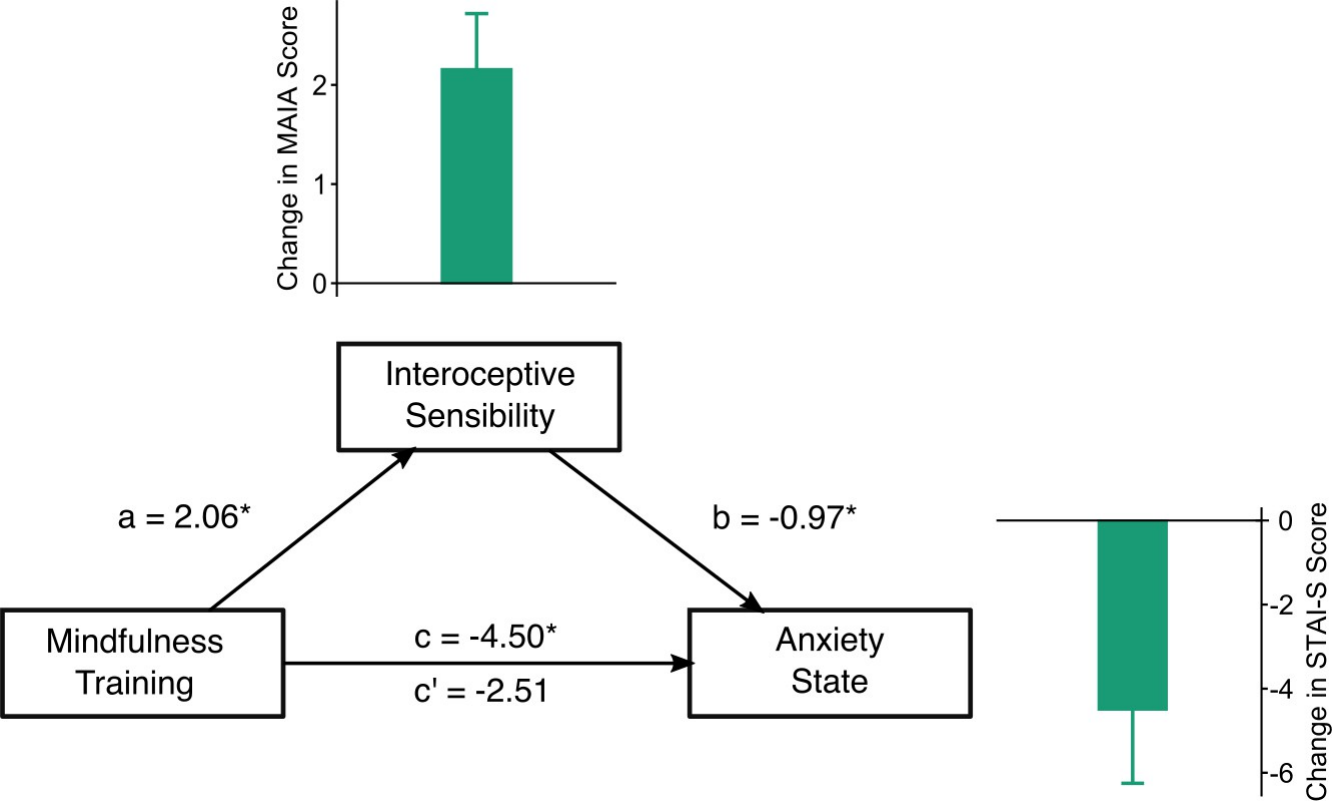
Mediation analysis. Diagram representing the mediation model in which MAIA change scores were found to mediate mindfulness-induced decreases in anxiety state scores (proportion mediated = 0.558). Unstandardized coefficients are depicted in each respective pathway on the graph. Insert plots are shown with the change in MAIA and Anxiety State scores after mindfulness training. a-path: effect of mindfulness training over interoceptive sensibility, b-path: effect of interoceptive sensibility over anxiety state, c-path: total uncontrolled effect of mindfulness training over anxiety state, c’-path: an indirect effect of mindfulness training over anxiety state after controlling for interoceptive sensibility. STAI-S: State and Trait Anxiety Inventory-State domain.

To investigate interoception as a potential mediator of MT-related anxiety reduction and test our second hypothesis, we performed a mediation analysis using the interoceptive sensibility change score as a mediator between the groups and anxiety state change scores (Fig 5), since these measures presented significant changes after training. We found that changes in MAIA scores mediated the mindfulness-induced decrease in anxiety state scores as shown by a significant indirect effect (B: -1.99, 95% CI: -5.69;-0.15, p = 0.03), and the loss of Mindfulness training effect on anxiety when controlling the MAIA change (c’: B = -2.51, 95% CI: -6.78;1.21, p = 0.22) compared to the uncontrolled effect (c: B = -4.50, 95% CI: -8.83;-0.48, p = 0.03), with a proportion mediated (c’/c) of 0.558 (Fig 5; S1 Table).

In the present study, we investigated whether a brief mindfulness training in healthy participants can lead to changes in interoception ability. Although interoceptive accuracy did not change after the mindfulness training, an important increase in interoceptive sensibility was observed along with a mediation of lower anxiety state scores. These findings bring new insights into the potential mechanisms involved in the therapeutic effects of brief mindfulness training.

Mindfulness meditation practices have been used as a complementary approach to regulating emotions by stabilizing attentional processes and improving self-awareness to facilitate more adaptive behaviors. The most used mindfulness technique directs attentional resources towards body sensation, especially the breath. With discipline and intention, mindfulness practitioners hold a curious, open, and non-judgmental attitude towards the experience that unfolds momentarily to cultivate a stable state of mind.

The interoceptive ability has been implicated in decision-making and emotional regulation, which seems to be influenced by meditation practices [1, 2, 42, 43]. Although previous studies suggest a relationship between interoception and mindfulness practices and between mindfulness practices and anxiety, little is known about the impact of a brief mindfulness-based training program on anxiety and interoception of healthy young adults, and the relationship between them.

In our study, interoceptive sensibility, as an overall domain of interoceptive ability, was enhanced only after mindfulness training (Fig 2B), partially confirming our first hypothesis. When analyzed separately, five out of eight MAIA subscales had higher scores after the mindfulness training, whereas no change was observed in the active control group (Fig 4). These subscales are related to attention regulation, emotional regulation, and body awareness. Between-group differences revealed that only the attention regulation subscale presented a significant difference (Fig 3). In this context, the change observed in the MAIA subscales in the present study may reflect beneficial, more adaptive mindful styles of attention [9]. Consistent with our findings, Bornemann and colleagues reported differential changes over time at the same MAIA subscales (attention regulation, emotional awareness, self-regulation, body listening and body trusting) after three months of contemplative training compared to a control group [44].

Additionally, the greatest changes after attentional training were seen in self-regulation, representing the ability to regulate psychological distress by attention to bodily sensation. Moreover, attention regulation, representing the ability to sustain and control attention to body sensations, showed the second-largest increase after mindfulness training [44]. In this study, we found a between-group difference only in the attentional regulation aspect of the interoceptive sensibility. Probably, a long-term intervention, such as that demonstrated by Bornemann and colleagues [44], would have a higher impact to differentiate groups and not only sessions by other self-regulatory domains beyond attention regulation.

We did not find differences in interoceptive accuracy within or between groups after mindfulness and active control training (Fig 2A). We also evaluated this interoceptive domain by analyzing blocks differentiated by time of the heart counting task and did not find any difference between or within groups (S1 Fig). These results differ from other findings in the literature that show a significant change after a mindfulness-based practice in accuracy but not in sensibility [45]. The possible explanation for such incongruence is the distinct length of the intervention (3 days x 8 weeks), the sort of exercises used in the intervention (attention to the breath x body scan), and the constructs used to assess interoceptive sensibility. On the other hand, our results are in line with recent studies showing that the practice of meditation is not associated with improved cardiac interoceptive awareness [31, 32]. Importantly, recent findings demonstrate that the objective domain of the interoception ability, such as IAc, is poorly related to measures of well-being, which are more associated with the subjective aspect of interoception [46]. Related to that, it is important to mention that depending on the attentional style and personal interpretation of an interoceptive cue, the emotional experience can differs [9]. In this way, MT may serve to override the commonplace goal of quickly triaging and responding to interoceptive signals, prioritizing instead a goal of exploration and tolerance for the multiple potential meanings of sensory experience. The consequence of such practice is the deconditioning and eventual extinction of interoceptive appraisal habits, which in contexts of anxiety can provide relief from catastrophization of interoceptive experience, a core feature of these disorders. By prioritizing exploration overreacting quickly to interoceptive experience, mindfulness practices seek to promote exploratory attitudes over the need to make rapid and accurate appraisals. As such, MT is somewhat orthogonal in its philosophy to clinical theory that seeks to relate interoceptive accuracy to mental health, instead indicating that interoceptive sensibility is a more relevant and tractable intervention target. Such theory is well-supported both by positive evidence relating measures of interoceptive sensibility to mental health [30, 47–49] and a corresponding lack of evidence relating interoceptive accuracy to wellbeing [30, 31, 50]. Moreover, evidence shows a weak correlation between interoceptive accuracy and sensibility, which indicates that a change in one domain is often not accompanied by changes in the other [10, 30, 44]. In fact, in our study there was no strong correlation between interoceptive accuracy and overall sensibility change for the mindfulness group (r_s_ = 0.21, p = 0.35) or for the control group (r_s_ = -0.02, p = 0.91).

In addition, many studies show that some aspects of interoception can be modified through attention training aimed at bodily sensation [1, 44, 45]. The neural and psychological implication of such change is the critical distinct activation and brain areas involved with interoceptive sensory processing and emotion regulation such as the anterior insula and the anterior cingulate cortex (ACC), leading to a reduction in anxiety symptoms followed by meditation practices [20, 51, 52].

Several studies have shown the therapeutic potential of mindfulness practices in reducing anxiety after long and brief interventions [53]. Recently, we showed that a brief mindfulness training regulates psychophysiological measures usually related to well-being and stress response [23]. Additionally, confirming our second hypothesis, our result presented in Fig 5 shows that the increase observed in interoception sensibility after a brief mindfulness-based training mediates the decrease in anxiety state scores (Fig 5). This effect can be explained by the acceptance aspect of the mindfulness attitude, which improves negative affectivity, stress, and stress-related health outcomes such as anxiety levels [13]. Moreover, the attentional style predominantly found in high anxiety-like states is characterized by an anticipatory mode of thinking, specially composed of self-referential thoughts aimed to predict situations [9, 36]. In this sense, it is plausible to suggest that attention regulation training based on a mindful attitude would lead to important changes in the attentional style of perceiving and interpreting body sensations.

While we present new insights into mindfulness-based training and interoception, this study has several caveats and limitations. First, the possibility of the type I error inflation due to multiple tests. Additionally, it is essential to consider the small sample size as one of the main limitations of this study, especially when using mediation analysis, which can present unstable behavior when the sample size is small. Furthermore, the lack of follow-up measures is also a limitation of the experimental design. In addition, a limitation of the heartbeat counting task must be taken into account, since participants could rely the counting of heartbeats in non-interoceptive cues or in estimates of heart rates rather than detecting the heartbeat sensation [54, 55]. Finally, a learning effect on the self-reported measures, especially MAIA, from the mindfulness-related terminology used in the intervention cannot be ruled out. However, our study found important evidence for understanding the acute psychological effects of brief mindfulness training. As far as we know, this is the first study to demonstrate the interaction between interoceptive sensibility and anxiety state after a brief mindfulness training. In addition, we show that mediation analysis is a valuable tool for studying psychological mechanisms induced by mindfulness practices.

## Conclusion

We observed that a brief mindfulness training focused on mindful awareness of bodily sensations and of the breath can improve interoception sensibility that mediates lower levels of anxiety. In other words, the awareness, as assessed by the self-reported interoceptive sensibility, derived from an audio-guided 30-minute mindfulness-based intervention during 3 consecutive days may mediate the decrease in students’ anxiety levels, as compared to an audio-guided general education plus coloring time-matched control. To date, this is the first randomized study with an active control group that addresses a mechanism by which a brief mindfulness-based training can potentially reduce anxiety in a healthy young non-clinical sample.

## Supporting information

S1 Fig Block analysis at the IAc (interoceptive accuracy) task according to the length of the counting period. No significant difference was found within (Mindfulness, n = 20: 25s: V = 66, p = 0.15, r = -0.33, 95% CI: -0.74; 0.14; 35s: V = 100, p = 0.87, r = -0.04, 95% CI: -0.48; 0.39; 45s: V = 79, p = 0.35, r = -0.22, 95% CI: -0.60; 0.25; Control, n = 20: 25s: V = 84, p = 0.67, r = -0.10, 95% CI: -0.58; 0.37; 35s: V = 77, p = 0.73, r = -0.08, 95% CI: -0.51; 0.40; 45s: V = 64, p = 0.13, r = -0.34, 95% CI: -0.69; 0.13) or between groups (25s: W = 181, p = 0.60, r = -0.08, 95% CI: -0.39; 0.25; 35s: W = 201, p = 0.989, r = 0.004, 95% CI: -0.321; 0.3050; 45s: W = 213, p = 0.738, r = 0.06, 95% CI: -0.267; 0.3620).
(TIFF)

S2 Fig Anxiety state scores before and after intervention. The figure shows box-plots before and after intervention with values of state anxiety inventory (SAI). Wilcoxon-signed rank test showed a significant intragroup difference for the Mindfulness group (V = 148, p = 0.03, r = 0.31, 95% CI: 0.027; 0.576) but not for the Control group was (V = 115, p = 0.72, r = 0.07, 95% CI: -0.245; 0.383). There was no Group x Time interaction on change scores and r effect size shows a low to medium practical significance (Post-Pre), (W = 252, z = 1.41, p = 0.16, r = 0.22, 95% CI: -0.127; 0.517).
(TIFF)

S1 Table Mediation model. Table with all causal mediation model coefficients showing the explanatory impact of Interoception sensibility change in decreased levels of anxiety after mindfulness training.
(DOCX)

S1 Checklist CONSORT 2010 checklist of information to include when reporting a randomised trial*. (DOCX)

S1 Protocol (DOCX)

S2 Protocol (DOCX)
