# Supplementary figures and images for: The impact of a brief mindfulness training on interoception: A randomized controlled trial
Source: PLoS One. 2022 Sep 7;17(9):e0273864. doi: 10.1371/journal.pone.0273864 (PMC9451078; doi:10.1371/journal.pone.0273864)

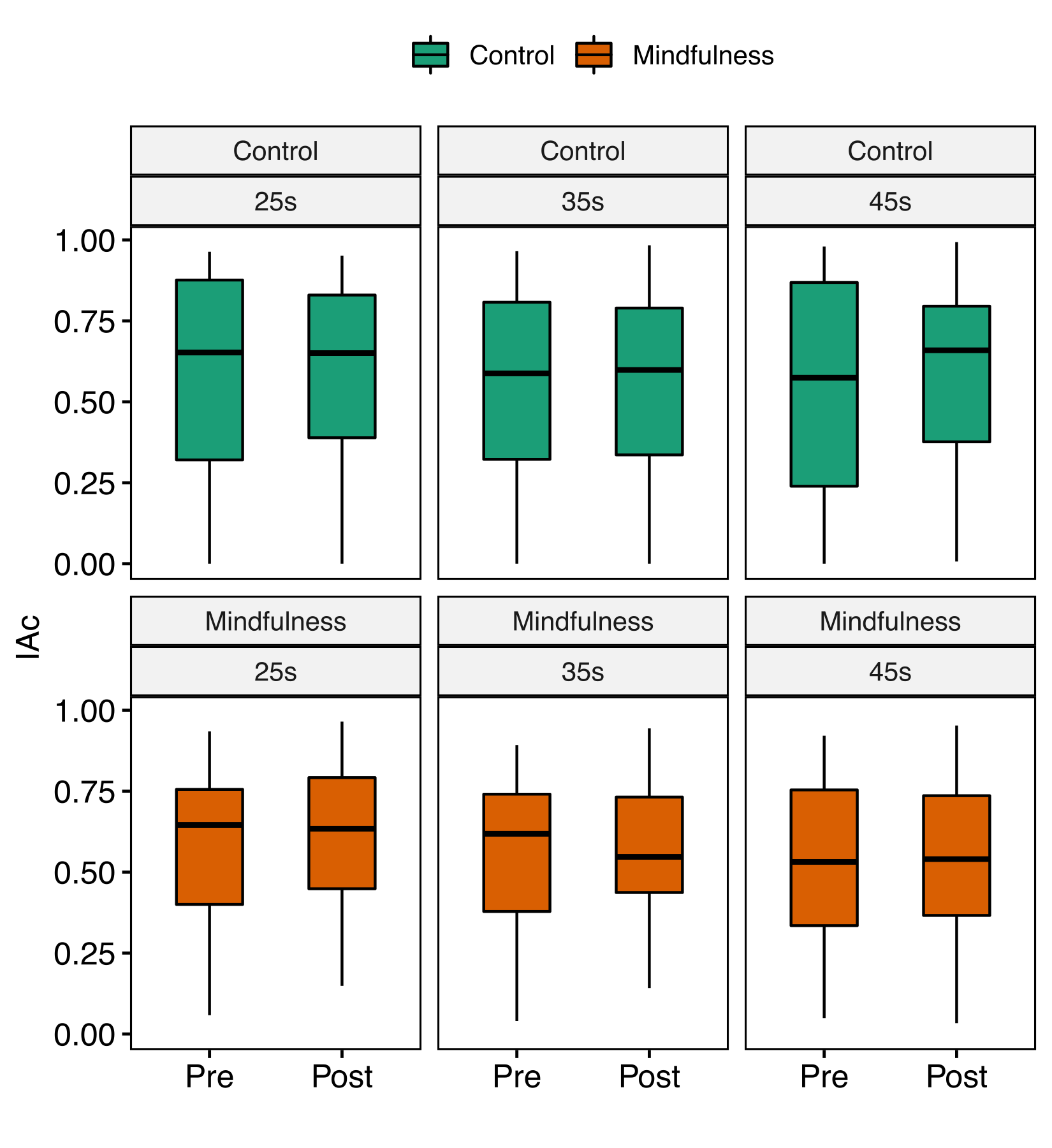

Supplement: S1 Fig — No significant difference was found within (Mindfulness, n = 20: 25s: V = 66, p = 0.15, r = -0.33, 95% CI: -0.74; 0.14; 35s: V = 100, p = 0.87, r = -0.04, 95% CI: -0.48; 0.39; 45s: V = 79, p = 0.35, r = -0.22, 95% CI: -0.60; 0.25; Control, n = 20: 25s: V = 84, p = 0.67, r = -0.10, 95% CI: -0.58; 0.37; 35s: V = 77, p = 0.73, r = -0.08, 95% CI: -0.51; 0.40; 45s: V = 64, p = 0.13, r = -0.34, 95% CI: -0.69; 0.13) or between groups (25s: W = 181, p = 0.60, r = -0.08, 95% CI: -0.39; 0.25; 35s: W = 201, p = 0.989, r = 0.004, 95% CI: -0.321; 0.3050; 45s: W = 213, p = 0.738, r = 0.06, 95% CI: -0.267; 0.3620). (TIFF) [file pone.0273864.s001.tiff]

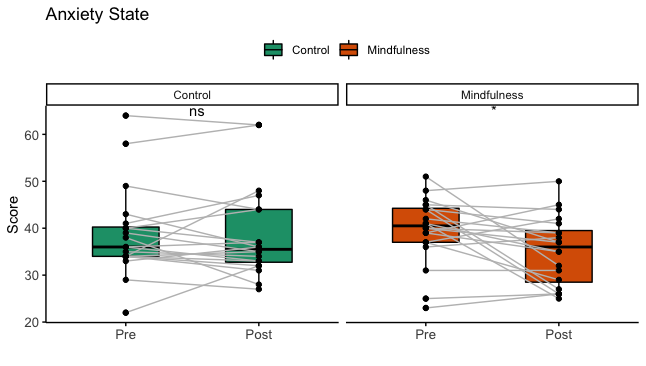

Supplement: S2 Fig — The figure shows box-plots before and after intervention with values of state anxiety inventory (SAI). Wilcoxon-signed rank test showed a significant intragroup difference for the Mindfulness group (V = 148, p = 0.03, r = 0.31, 95% CI: 0.027; 0.576) but not for the Control group was (V = 115, p = 0.72, r = 0.07, 95% CI: -0.245; 0.383). There was no Group x Time interaction on change scores and r effect size shows a low to medium practical significance (Post-Pre), (W = 252, z = 1.41, p = 0.16, r = 0.22, 95% CI: -0.127; 0.517). (TIFF) [file pone.0273864.s002.tiff]
