# Supplementary material for: The impact of a brief mindfulness training on interoception: A randomized controlled trial
Source: PLoS One. 2022 Sep 7;17(9):e0273864. doi: 10.1371/journal.pone.0273864 (PMC9451078; doi:10.1371/journal.pone.0273864)
Supplement: S1 Table — Table with all causal mediation model coefficients showing the explanatory impact of Interoception sensibility change in decreased levels of anxiety after mindfulness training. (DOCX) [file pone.0273864.s003.docx]

**S1 Table:**

|  | **B** | **SE** | **z** | **Bootstrap 95% CI** | | **p-value** |
| --- | --- | --- | --- | --- | --- | --- |
|  |  |  |  | **Lower** | **Upper** |  |
| **a** | 2.06 | 0.84 | 2.45 | 0.41 | 3.72 | 0.01 |
| **b** | -0.97 | 0.39 | -2.48 | -1.66 | -0.11 | 0.01 |
| **c’** | -2.51 | 2.07 | -1.21 | -6.79 | 1.31 | 0.22 |
| **Indirect effect** | -1.99 | *NA* | *NA* | -5.69 | -0.15 | *NA* |
| **Total effect** | -4.50 | 2.13 | -2.11 | -8.83 | -0.48 | 0.03 |

B = unstandardized estimate, SE = standard error of the mean, a = Group → State mindfulness pathway, b = State mindfulness → MAIA pathway, c’ = Direct effect (Group → MAIA, without accounting for State mindfulness effect), *NA =* Not applied.
