# Supplementary material for: The impact of a brief mindfulness training on interoception: A randomized controlled trial
Source: PLoS One. 2022 Sep 7;17(9):e0273864. doi: 10.1371/journal.pone.0273864 (PMC9451078; doi:10.1371/journal.pone.0273864)
Supplement: S1 Protocol — (DOCX) [file pone.0273864.s005.docx]

FEDERAL UNNIVERSITY OF RIO GRANDE DO NORTE

BRAIN INSTITUTE

**Official Title:** IMPACT OF MINDFULNESS-BASED PRACTICES ON STRESS RESPONSE, EMOTIONAL REGULATION, INTEROCEPTIVE AND ATTENTIONAL SKILL IN HEALTHY INDIVIDUALS

**Approval number:** 1.761.383

**CAAE:** 55193416.4.0000.5537

**Principal Investigator:**

Maria Bernardete Cordeiro de Sousa

**Co-Investigators:**

Geissy Lainny de Lima Araújo

Geovan Menezes de Sousa Junior

Thatiane M. A. S. Mendes

Marcelo Demarzo

Norman Farb

Draulio B Araujo

**Main study centres**

Brain Institute

Federal University of Rio Grande do Norte- UFRN

Av. Senador Salgado Filho, 3.000, Campus Universitário, Lagoa Nova
CEP: 59078-900, Caixa Postal 1524, Natal/RN

**Financial support**

CNPq 484972/2013‑8 and 306051/2017-6.

**Abstract**

Currently, young adults are exposed to work and study overload and, therefore, often end up developing stress-related illnesses. Mindfulness-based stress reduction programs (MBSR) have been carried out in the most diverse contexts, being treated as possible therapies for depression, anxiety, chronic pain, and chronic stress. Originally created as an eight-week program, it is now being adapted for brief interventions leading to greater adherence to the activities and to the program itself. The relationship of the practices with improvement of attention on daily activities has been demonstrated. However, the interoceptive component that is directly trained as part of mindfulness-based practices, and it is related to well-being and greater capacity for self-regulation is not well understood. In this sense, the present study seeks to understand the relationship between brain activity, emotional regulation, attentional and interoceptive skills after a brief mindfulness-based intervention. To address this aim, blood and saliva cortisol levels will be assessed. Moreover, cognitive and attentional tasks will be performed in addition to evaluation of interoception, anxiety, stress and mindfulness as a trait and as a state.

**1. Introduction**

In the las decades, the literature has shown the use of new approaches with a mind-body integrative focus in order to prevent or treat illnesses that have increased with the lifestyle adopted in current societies, such as anxiety, depression and stress (ESCH et al. ., 2007; MARCHAND, 2012; UEBELACKER; BROUGHTON, 2016; WANG et al., 2014). Stress can be characterized as any disturbance to the body's homeostasis provided by exteroceptive stimuli (eg, predator smell, visual information) or interoceptive stimuli (blood volume, osmolarity) (ULRICH-LAI; HERMAN, 2009). In the social and psychological sphere, a survey carried out in the United States showed that the main triggering factors for stress are the economic situation, employment, family, and health status (APA, 2014). Stress is manifested in individuals of all ages, especially in young adults who are often subjected to study and work routines, in parallel, leading to a biopsychosocial overload.

The general adaptation syndrome or stress response was first described by Hans Selye in 1936 (Selye, 1936) and aims to maintain the physiological integrity of the organism. Therefore, this response is extremely necessary for survival if it is maintained at optimal levels, its absence or exacerbation being responsible for important physiological disorders and inappropriate responses to a specific demand (MCEWEN; WINFIELD, 2010). It is primarily mediated by the Hypothalamus-Pituitary-Adrenal (HPA) axis through a cascade of cellular events that lead to the release of hormones into the bloodstream. One of the main markers of HPA axis reactivity to stress is the hormone cortisol, which is released by the biological clock in a circadian rhythm and through stressful situations to the organism's homeostasis (HAUS, 2007; SELMAOUI; TOUITOU, 2003). This reactivity can be measured using various techniques, including investigation using blood plasma, urine, and saliva. From a stressful stimulus, there is activation of neurons located in the paraventricular nucleus (PVN) of the thalamus, which produce and secrete the corticotropic hormone releasing factor in the portal-pituitary system. Subsequently, there is the production and release, from adenohypophysis, of the adrenocorticotrophic hormone (ACTH) that, through the bloodstream, reaches the adrenal gland cortex, promoting the production and release of glucocorticoids, such as cortisol. Cortisol acts by negative feedback on the hypothalamus and pituitary in such a way that, when reaching high concentrations in the blood, it inhibits the release of factors that promote its production (SANDERS, 1983; SAPOLSKY; ROMERO; MUNCK, 2000; STRATAKIS; CHROUSOS, 1995; TSIGOS; CHROUSOS, 2002; ULRICH-LAI; HERMAN, 2009). In addition to the activation of the HPA axis, there is an important regulation of the autonomic nervous system (ANS) during the stress response, such as, for example, the change in cardiac activity, mediated mainly by the release of catecholamines, which also act on the PVN nucleus of the thalamus and limbic regions (GOLDSTEIN, 2003; KOPIN, 1995; ULRICH-LAI; HERMAN, 2009). In addition, neuromodulators such as BDNF (brain-derived neurotrophic factor) and the expression of its receptors are also affected in cases of acute or chronic stress, an effect observed in studies with rodents and humans (LICINIO; WONG, 2002; MURAKAMI et al., 2005; TAKAHASHI et al., 2000). BDNF is mainly related to synaptic plasticity and neurogenesis, and alterations in its release or in the expression of its receptors can cause deficits in important basic processes such as the acquisition of a new memory or development of psychiatric disorders (ALLEVA; SANTUCCI, 2001; BERTON et al. al., 2006; HORCH et al., 1999; MURAKAMI et al., 2005; TAKAHASHI et al., 2000). The brain's response to stressful stimuli (non-physical or psychogenic stimuli) is based on previous experience from the activation of previously established neural circuits or on innate programs of the organism. These reactions are usually processed in the forebrain, involving the limbic system and may occur in anticipation or in response to a stressful stimulus, for review see (ULRICH-LAI; HERMAN, 2009).

**Mindfulness-based programs**

The search for ways to reduce the impact of routine and social pressure on health led to the development of preventive and therapeutic alternatives for situations of stress. One is the use of systematized meditative practices over eight weeks known as the Mindfulness Based Stress Reduction program (MBSR).

Mindfulness-based programs began in a clinical setting in the 1970s through the development of a program for managing stress and chronic pain (KABAT-ZINN, 1990; KABAT-ZINN; LIPWORTH; BURNEY, 1985). Currently, there are several modalities of mindfulness-based programs aimed at specific pathologies or groups. Among the best known are MBSR, MBCT (Mindfulness based cognitive therapy) and MBRP (Mindfulness based relapse prevention). Overall, mindfulness-based stress reduction practices form a well-established training program over 8 (eight) weeks. In this program, activities are developed that use meditative practices as a tool aimed at refining the attention and self-awareness of the practitioner (KABAT-ZINN, 1990). What is cultivated in this practice is a present moment awareness, with openness, curiosity and a non-judgmental attitude, where the attentional focus is directed to the observation of the experience of thoughts, bodily sensations and emotions (HOLZEL et al., 2011; KABAT-ZINN, 1990).

Training effects have been demonstrated as efficient in the treatment of different diseases with important outcomes such as structural and functional changes of the central nervous system (CNS) and, consequently, in the expression of the individual's behavior. Disorders such as anxiety (KOCOVSKI et al., 2015), depression (HOFMANN et al., 2010), substance abuse (BOWEN et al., 2007, 2014) and eating disorders (GODSEY, 2013; O'REILLY et al., 2014) are among the most studied in the context of the application of mindfulness as a therapy. In addition, rehabilitation after breast cancer (HUANG et al., 2015; WÜRTZEN et al., 2015) and cases of chronic pain (BAWA et al., 2015; COUR; PETERSEN, 2015; KABAT-ZINN; LIPWORTH; BURNEY, 1985) also characterize a large part of the studies carried out in the last 30 years.

With the diversity of works found in the literature about the application of mindfulness-based programs, there are three main lines where the studies are concentrated: attention training, benefiting people with attention deficit hyperactivity disorder or similar condition (ZYLOWSKA DEBORAH ACKERMAN MAY H YANG JULIE L FUTRELL NANCY L HORTON T SIGI HALE; PATAKI; SMALLEY, 2008), better emotional regulation from the recognition of automatic modes of thoughts or feelings in people with depression (KUYKEN et al., 2015), and o development of self-knowledge and improved resilience in the face of stressful situations in people with some mental disorder (DAVIS; SETH KURZBAN, 2012).

MBSR (specifically aimed at reducing stress) has been widely studied in healthy individuals or with some disease and its effect has been demonstrated on the neuroendocrine axis with short and long-term interventions (BROWN; WEINSTEIN; CRESWELL, 2012; CHIESA; SERRETTI, 2009; CRESWELL et al., 2014; DECKRO et al., 2002; O'LEARY; O'NEILL; DOCKRAY, 2015)

Creswell et al. in 2014 demonstrated that a 3-day MBSR exercise practice improved participants' perception of stress when compared to a control group. In this study, participants underwent the social stress test (TSST - Trier Social Stress Test) after the interventions and it was found that people who have a more pronounced mindfulness trait before the intervention have lower neuroendocrine axis reactivity to acute stress. However, compared to the control group, the group undergoing mindfulness practice had, in general, an increase in the level of salivary cortisol (CRESWELL et al., 2014). In fact, the mindfulness trait seems to influence cortisol levels and the individual's relationship with negative emotions, with those with a lower trait being more prone to emotional dysfunctions and those with a higher trait showing better adaptation to a stressful stimulus (BROWN; WEINSTEIN; CRESWELL, 2012; DAUBENMIER et al., 2014).

In a 6-week mindfulness-based program in students, was observed lower levels of perceived stress, anxiety, and depression (DECKRO et al., 2002).

In a systematic review about the relationship between mindfulness practice and cortisol levels, the authors report the impossibility of reaching a common conclusion about the results found. This is due to lack of randomization or methodological problems found in the studies. (O’LEARY; O’NEILL; DOCKRAY, 2015).

**Mindfulness, emotional regulation, attentional and interoceptive ability**

Recently, some studies have demonstrated structural and functional changes observed in the brain of experienced meditators (HASENKAMP; BARSALOU, 2012; HÖLZEL et al., 2007; KANG et al., 2013; KILPATRICK et al., 2011; KURTH et al., 2015; LAZAR; LAZAR et al., 2005). Generally, brain areas that exhibit differentiated activity in meditative practitioners consist of cortical and subcortical regions involved in the limbic system. They are the prefrontal cortex, a region related to metacognition, planning and judgment; hippocampus, an area related to memory processing; anterior and orbitofrontal cingulate cortex, areas related to the perception of the “self”, attentional and emotional regulation; insula, region related to interoception; amygdala, region related to emotional processing, in addition to areas involved with inter-hemispheric communication such as the corpus callosum and the superior longitudinal fasciculus for review, see (TANG; HÖLZEL; POSNER, 2015b). Interestingly, regions of the limbic system seem to be related to the regulation of the paraventricular nucleus of the hypothalamus, an important site that constitutes the HPA axis. Thus, changes in activation of limbic regions can lead to physiological differences in the response to some stressful event (ULRICH-LAI; HERMAN, 2009).

One of the main components of mindfulness-based activities is attention regulation (BISHOP et al., 2004). From the implementation of these practices, changes have been observed in the subcomponents of the attentional system, such as orienting, alerting and monitoring in different contexts (MALINOWSKI, 2013; TANG; HÖLZEL; POSNER, 2015b).

Mindfulness practices involve focused attention, when it uses a fixed focus during its execution and is present mainly at the beginning of practices, and open monitoring, when the individual develops the ability to naturally move their attention between the elements of the experience (MALINOWSKI, 2013). Overall, studies demonstrate better performance on attentional tests in experienced meditators and this is correlated with cortical areas directly involved in the attention process. (MALINOWSKI, 2013; TANG; HÖLZEL; POSNER, 2015b). Jha et al. in 2007 demonstrated better performance in the Attention network test (ANT) of individuals who underwent an 8-week MBSR and individuals who participated in a month-long retreat and performed focused attention practices and open monitoring, corroborating other contemporary studies to this one (JHA; KROMPINGER; BAIME, 2007; TANG et al., 2007).

Regarding the attentional component, using electroencephalography (EEG), Fan et al. demonstrated an increase in the alpha wave potential in frontoparietal regions during an attention test after the practice of mindfulness-based exercises. The increase in the potential of these waves, in this region, seems to be associated with a better cognitive performance, reflected in a shorter reaction time to the stimulus, in addition to a smaller number of errors throughout the task (FAN et al., 2014). In addition, a reduction in frontal alpha asymmetry has also been reported after an 8-week mindfulness program. This result seems to be related to a better emotional regulation capacity involving greater well-being and less reactivity to emotionally negative situations (ZHOU; LIU, 2016).

Interoception or interoceptive ability can be described as the individual's ability to become aware of the signals originated from the body itself (FARB et al., 2015). This ability has been related to greater well-being and better self-regulation, considering that it is necessary for the perception of internal events that are important for survival (FARB et al., 2015; LIOTTI et al., 2001). In a recent study, an interesting change was observed in the self-report of personal aspects such as attention to the body after 3 months of contemplative practices (BORNEMANN et al., 2015). Along with changes in self-report, the change in interoceptive attention was measured through an instrument developed for this purpose called Multidimensional Assessment of Interoceptive Awareness (MEHLING et al., 2012).

Farb et al. in 2013 demonstrated that mindfulness practice leads to greater cortical activity in regions related to interoception, such as the anterior portion of the insula (FARB; SEGAL; ANDERSON, 2013). Considering that most mindfulness practices use attention focused on internal events, the interoceptive component as a benefit of the practices must be considered.

However, there is no study in the literature that demonstrates the effects of a brief mindfulness-based intervention on cortical activity, affective measures and interoceptive ability.

**2. Objectives**

General:

• Assess the impact of a brief training on the stress response, interoception and well-being on healthy individuals.

Specific:

• Verify the relationship between mindfulness trait and hypothalamic-pituitary-adrenal axis reactivity.

• Analyze the neuroendocrine response to a stress event after a brief mindfulness-based intervention.

• Assess the impact of mindfulness practice (3 days) on the attention system, autonomic nervous system, and interoceptive ability.

• Check the influence of the practice on self-report measures of stress, anxiety, and affect

• Assess participants' electroencephalographic activity before and after the proposed intervention, during a sustained attention test, an interoception test, and during the stress test.

**3. Materials and Methods**

Participants

Fifty young adults of both sexes, aged between 18-35 years will be recruited through wide dissemination on the campus of the Federal University of Rio Grande do Norte-UFRN. The sample size was determined with G * Power of the F test family (RM-ANOVA with intra-between interaction), considering a moderate to high effect size (f = 0.3) at α = 0.05 and 80% of power, as well as 60% correlation between repeated measures and considering loss of participants during the study being necessary at least 40 participants to attend these parameters.

Participants will be interviewed in advance and will complete a series of questionnaires to verify their eligibility for the study. Upon being elected to the study, they will be randomized into two groups proposed in this study.

**Inclusion criteria:**

• Age between 18 and 35 years old.

• Have no clinical psychiatric diagnosis (schizophrenia, major depression, bipolar disorder, social anxiety disorder).

• Do not use psychotropic, beta-blocker or anti-inflammatory drugs at the time of the study.

• Have no prior experience with mindfulness-based practices.

• Sign the written consent

**Exclusion Criteria**

• Absence of one day of the study

• Have an infectious disease during the study

• Body mass index (BMI) above 30.

**Training**

Based on previous studies, a brief intervention will be performed for 3 days for 30 minutes a day, involving practices contained in the mindfulness program for stress reduction. The exercise used in the study will be a practice of attention focused on the sensations of breathing that will be recorded by a qualified professional. The participant will come to the laboratory during the morning for 3 consecutive days and must stay in a room dedicated to practices throughout the procedure. The control group will spend the same time in a room designated for the study and will be subjected to a task of coloring figures during the same time as the experimental group. The study steps are specified in the diagram below:

Figure 1: Timeline of assessments throughout the study of groups 1 and 2. EEG: Electroencephalography.

In a schematic way, the evaluation with the EEG will be carried out as follows:

Figure 2: Schematic showing the activities to be performed during data acquisition using electroencephalography (EEG).

**Stress Induction and Assessment of Emotional Regulation**

The stress test to be used is the Mannheim multicomponent stress test (MMST) (KOLOTYLOVA et al., 2010). The MMST is a multi-component test that involves emotional, auditory, and arithmetic stimulation for 5 minutes. The response of the HPA and SNA axis seems to be like that obtained with the TSST (Trier Social Stress Test), the standard test used to verify the stress response in laboratory studies. (REINHARDT et al., 2012). In the first 3 minutes of the test, the participant is presented with several images with positive, negative, and neutral emotional valences and is asked to categorize the image into these valences on a scale of 1-7. A total of 52 images will be displayed. After that, a white noise ranging from 78-98dB is presented and the participant is asked to perform an arithmetic test according to what appears on the computer screen in front of him. This test is the PASAT (Paced Auditory Serial Addition Test) in which the participant must add the last digit shown to the one immediately before, typing the sum of both digits on the keyboard in front of him.

**Biochemical and physiological assessment**

**Electroencephalography (EEG), Electrocardiogram (ECG) and galvanic skin response (GRS):**

Electroencephalography will be performed with a 64-channel BrainAmp DC device (Brain Products GmbH, Munich, Germany), using ActiCap active electrodes (Brain Products GmbH, Munich, Germany). The ECG and GSR electrodes will be connected to BrainAmp ExG (Brain Products GmbH, Munich, Germany). The EEG data will be analyzed using Matlab software using the EEGLAB toolbox (DELORME; MAKEIG, 2004).

**Saliva:**

Saliva samples will be collected before the start of the stress test and 15, 30 and 60 minutes after its end. The samples will be collected in an appropriate device - Salivettes (Sarstedt, Germany) and later processed, centrifuged at 3000 RPM, for 15 minutes and stored at -30º C until further dosing.

**Dosage of salivary cortisol**

Salivary cortisol dosages will be performed from samples collected on day 3 of the protocol. They will be made using commercial dosage kits from the company DRG Instruments GmbH (Germany).

The basic principle used by the kit is the competition ELISA, in which a microplate is sensitized with monoclonal antibodies (IgG) to cortisol. The reading of samples will be performed by spectrophotometry.

On day 1 of the protocol, the participant will answer the pre-intervention questionnaires and a blood sample will be collected for subsequent analysis of cortisol and BDNF.

**Blood collection:**

Study participants will undergo venipuncture of 20 ml of blood after fasting for 8 hours before and after the intervention proposed in this project. They will be instructed to have a peaceful night's sleep and to attend the collection point at the agreed time. Blood collection will be carried out in the non-dominant arm of each participant, in the morning (between 8:00 and 9:30 am) after 45 minutes of the participant's rest. Peripheral blood samples will be collected without anticoagulant (10 mL) to study the hematological, lipid, hepatic and renal profiles and the endocrine marker (cortisol) and BDNF.

**Dosage of plasma cortisol**

Cortisol levels will be determined in duplicate by the immunoenzymatically method (ELISA). Coefficients of variation will be calculated and must be less than 20%. The basic principle used by the kit is the competition assay, in which a microplate is sensitized with monoclonal antibodies (IgG) to cortisol. Unknown samples (from participants) compete with cortisol linked to a peroxidase enzyme for the cited antibodies. After an incubation period, unbound components are washed away. A substrate (tetramethylbenzidine) is added, and it will oxidize the cortisol-bound enzyme producing a color change. With the addition of a stop solution, another color change occurs and then a spectrophotometric reading will be taken using a filter with an adequate wavelength. It is noteworthy that using this type of ELISA, the higher the optical density, the lower the amount of hormone present in the sample, the opposite being true.

**BDNF dosage**

2mL aliquots of plasma will also be separated after blood collection, for BDNF analysis, through the enzyme immunoassay technique (ELISA), using commercial kits.

**Autonomic Nervous System Assessment**

The measurement of the modulation of the Autonomic Nervous System (ANS) on the heart will be performed using the Heart Rate Variability (HRV) technique that uses the RR intervals of the electrocardiogram (ECG) captured and recorded for one hour with the volunteers in awake with electrodes connected to BrainAmp ExG (Brain Products GmbH, Munich, Germany).

Time and frequency domain measurements (HF (High frequency), LF (Low frequency), VLF (Very low frequency) and Ratio (LF/HF) will be used to represent cardiac vagal activity.

**Interoceptive evaluation**

In addition to applying the scale for interoceptive assessment (MAIA) described above, we will carry out an objective assessment based on previous studies (KOCH; POLLATOS, 2014; SCHANDRY, 1981).

The heartbeat count test will be used to verify the cardiac accuracy that reflects the level of interoceptive sensitivity of the volunteers. This test is widely used in the literature and cardiac interoception is representative of body interoception. The test consists of silent counting without tactile handling of the number of heartbeats perceived during three periods of time 25s, 35s and 45s, which are randomly combined, interspersed by 30s (SCHANDRY, 1981). Thereafter, the perceived number of beats is compared with the actual number of beats, resulting in the level of cardiac accuracy. The counting protocol with the three time periods interspersed by 30s will be repeated three times, and the mean of the two closest cardiac accuracy values ​​will be considered. The repetition of this procedure on different days will be performed to ensure the reproducibility of the level of interoceptive accuracy of the volunteers. Volunteers will be classified as having high and low interoceptive sensitivity based on the sensitivity criterion above or below 75% accuracy, respectively.

**Sustained Attention**

To verify the attentional component, we will perform a sustained attention response task. The test will always be at the same time to avoid influences from the circadian rhythm (RICCIO et al., 2002; VALDEZ et al., 2005). This test basically consists of the participant's use of the dominant hand to press the space bar on the keyboard in front of them when any digit, except the digit 3, appears on the computer screen. Thus, the participant must inhibit his response during the appearance of the digit 3 (ROBERTSON et al., 1997). The number of correct answers and response time will be measured in the two session that will be performed before and after the intervention. The e-prime software will be used for the programming and application of the test.

**Questionnaires**

The questionnaires described below will be applied before, during and after the interventions.

**Multidimensional Assessment of Interoceptive Awareness - MAIA**

Questionnaire designed to measure the ability to identify emotions and interoceptive bodily sensations (MEHLING, 2012). Items fall into some domains that refer to the following factors:

1) Awareness of bodily sensations.

2) Emotional reaction and attentional response to bodily sensations.

3) Emotion regulation capacity.

4) Consciousness of Mind-Body Integration.

5) Confidence in bodily sensations.

The instrument contains 32 items and has an internal consistency of 0.82. We will work on validating this questionnaire for the Brazilian population and using the version translated into Portuguese (MAIA-VB2).

Mindful Attention Awareness Scale (MAAS)

Scale that assesses the level of Mindfulness, consisting of 15 items, on a six-point Likert scale ranging from (1) almost always to (6) almost never. The scale is one-dimensional, so all items measure Mindfulness. We will use the Brazilian version of the questionnaire (ATANES et al., 2012) and we will work on validating this version.

**FFMQ- Five Facets of Mindfulness Questionnaire - (FFMQ)**

This scale assesses the level of Mindfulness and is composed of 39 items on a five-point Likert scale ranging from (1) never or rarely true to (5) almost always or always true. On this scale, the concept of Mindfulness is divided into five components, they are:

(1) Observing, which includes noticing or being aware of internal and external experiences, such as sensations, cognitions, emotions, sights, sounds and smells.

(2) Describe, which refers to characterizing internal experiences through words.

(3) Acting with awareness, which refers to being aware of the activities of the moment and can be contrasted with mechanical behavior while attention is focused on something else, known as autopilot.

(4) Non-judgment of internal experience, which refers to not taking an evaluation and judgmental stance in relation to thoughts and feelings.

(5) Non-reactivity to internal experience, which refers to the tendency to allow thoughts and feelings to come and go without being affected or taken over by them (BAER et al., 2006). We will use the Brazilian version of the questionnaire (BARROS et al., 2014).

**State Mindfulness Scale - (SMS)**

This scale aims to assess levels of mindfulness related to the state of consciousness (TANAY; BERNSTEIN, 2013). It was developed based on previous mindfulness trait scales and seeks to complement the mindfulness assessment at the time of its application. It is a scale that contains 21 items where the individual will have to answer the questions with a graduation from “nothing” to enough”. We will work on validating this scale for the Brazilian population.

**Perceived Stress Scale**

The Perceived Stress Scale - PSS (COHEN, 1983) is one of the most cited instruments in the literature for stress estimation. PSS assesses stress under three aspects: presence of specific agents that cause stress, physical and psychological symptoms of stress and general perception of stress, regardless of its causative agent (for example, in item 3: "In the past month, how often have you did you feel nervous or stressed?”). According to the authors, other instruments available in the literature intend to estimate the specific impact of some stressful factors, which limits their results as stress-related events can vary between individuals. In this study, we will use the short version of this scale translated and validated for the Brazilian population (DIAS et al., 2015)

**Positive and Negative Affect Schedule - (PANAS)**

This questionnaire consists of a set of words that describe different feelings and emotions arranged in two mood scales with 10 items each (WATSON; CLARK; TELLEGEN, 1988). Each item is scored from 1 to 5 points (1 = "very slightly or not at all" to 5 = "extremely") to indicate the extent to which respondents felt feelings and emotions during the weeks prior to the interview. We will use the version validated in Portuguese by (GALINHA; PAIS-RIBEIRO, 2005).

**Amsterdam Resting-state Questionnaire- ARSQ**

This instrument aims to assess the relationship with thoughts in a state of rest. Developed by Diaz et al., 2013 and improved in 2014 (ALEXANDER DIAZ et al., 2014; DIAZ et al., 2013), it seems to be of great value when applied to participants undergoing contemplative practices. Version 2.0 to be used in this study consists of 54 items that should be answered on a scale of 1-5 that varies from “completely disagree” to “completely agree”.

**State-Trait Anxiety Inventory - (STAI)**

This inventory seeks to assess through two parts, each containing 20 statements, anxiety state and trait. It was developed by Spilberg et al (1970) and translated and validated for the Brazilian population by Biaggio and Natalício (1979). The participant must respond on a scale of 1-4 and the total score for each scale ranges from 20 to 80, with higher values ​​being indicative of higher levels of anxiety.

**Statistical analysis**

Initially, Kolgomorov-Smirnov normality tests will be applied to determine if the data obey a normal distribution. Once this prerequisite is met, Student's t test (parametric variables) or Mann Whitney U test (non-parametric variables) will be applied to detect the occurrence of significant differences within group and ANOVA to evaluate between groups differences. Spearman's correlation test will also be applied to test the occurrence of correlations between variables (anxiety, affect, stress and mindfulness, trait and state).

An exploratory and confirmatory factor analysis will be performed to verify the domains and association between the factors existing in the questionnaires. The internal consistency of the factors will be verified using Crombach's Alpha and only saturation values ​​greater than 0.4 for the eigenvalues ​​will be computed for the item's participation in the investigated domain.

For the analysis of plasmatic and behavioral markers, correlation tests and multivariate analysis will be used.

For all tests, the significance level (p value) less than or equal to 0.05 will be considered.

**Risk and Benefit Assessment:**

Risk:

During blood collection, the person may experience dizziness or a slight drop in pressure. Any discomfort will be minimized by a suitably qualified professional and any procedure necessary to maintain the participant's health and well-being will be carried out. When exposed to the social anxiety test, the participant may feel nervous and show symptoms of anxiety and stress. However, there will always be a professional with the individual providing any necessary support.

Benefits:

Based on previous studies, mindfulness-based practices for stress reduction seem to modify the stress response via the hypothalamic-pituitary-adrenal axis to allow a better adaptation to a stressful stimulus. In addition, symptoms of anxiety, depression and perceived stress are significantly improved after the practice of these activities. Additionally, the low cost and easy execution of these practices support their use in daily life to reduce stress in clinical and non-clinical populations. Aiming at a possible intervention with these characteristics at the educational level and in the public health system, the results obtained in this study will bring to light new understandings about its functioning and application.

**Ethical aspects**

The study will be carried out according to the "Declaration of Helsinki" (1974), revised in Tokyo (1975), Venice (1983), Hong Kong (1989), South Africa (1996) and Scotland (2000). Resolution CNS 196/196. For all volunteers, the consent form will be read, providing information on the justification, objectives, procedures, risks and benefits of the study to which they are being invited to participate. All subjects will provide written informed consent before participation. Subjects who meet inclusion criteria will be invited to participate in the trial. All patients are free to withdraw at any time from the protocol interventions without giving reasons and without prejudicing their further treatment. To all patients will be guaranteed the right to receive information and clarifications regarding any doubts that may arise during the experimental protocol and updated information about the study. The research team will preserve the confidentiality of patients taking part in the study. Patient’s identities will not be revealed, as well as that all information provided by them will be kept confidential.

**References**

ALEXANDER DIAZ, B. et al. The ARSQ 2.0 reveals age and personality effects on mind-wandering experiences. **Frontiers in Psychology**, v. 5, n. APR, p. 1–8, 2014.

ALLEVA, E.; SANTUCCI, D. Psychosocial vs. “physical” stress situations in rodents and humans: Role of neurotrophins. **Physiology and Behavior**, v. 73, n. 3, p. 313–320, 2001.

ATANES, A. et al. Validade e confiabilidade da “ Escala de Atencão Plena e Consciência ” ( MAAS ) e “ Questionário das Cinco Facetas de Mindfulness ” ( FFMQ ) entre profissionais da Atenção Primária à Saúde. **Revista Brasileira de Medicina de Família e Comunidade**, p. 2012, 2012.

BAER, R. A. et al. Five Facet Mindfulness Questionnaire. **Assessment**, v. 13, p. 27–45, 2006.

BARROS, V. V. DE et al. Validity evidence of the Brazilian version of the Five Facet Mindfulness. **Psicologia: Teoria e Pesquisa**, v. 30, n. 3, p. 317–327, set. 2014.

BAWA, F. L. M. et al. Does mindfulness improve outcomes in patients with chronic pain? Systematic review and meta-analysis. **The British journal of general practice : the journal of the Royal College of General Practitioners**, v. 65, n. 635, p. e387–e400, 2015.

BERTON, O. et al. Essential role of BDNF in the mesolimbic dopamine pathway in social defeat stress. **Science (New York, N.Y.)**, v. 311, n. 5762, p. 864–8, 2006.

BISHOP, S. R. et al. Mindfulness: A Proposed Operational Definition. **Clinical Psychology: Science and Practice**, v. 11, n. 3, p. 230–241, 11 maio 2004.

BORNEMANN, B. et al. Differential changes in self-reported aspects of interoceptive awareness through 3 months of contemplative training. **Frontiers in Psychology**, v. 6, n. JAN, p. 1–13, 2015.

BOWEN, S. et al. The role of thought suppression in the relationship between mindfulness meditation and alcohol use. **Addictive behaviors**, v. 32, n. 10, p. 2324–8, out. 2007.

BOWEN, S. et al. Relative Efficacy of Mindfulness-Based Relapse Prevention, Standard Relapse Prevention, and Treatment as Usual for Substance Use Disorders. **JAMA Psychiatry**, v. 71, n. 5, p. 547, 1 maio 2014.

BROWN, K. W.; WEINSTEIN, N.; CRESWELL, J. D. Trait mindfulness modulates neuroendocrine and affective responses to social evaluative threat. **Psychoneuroendocrinology**, v. 37, n. 12, p. 2037–2041, 2012.

CHIESA, A.; SERRETTI, A. Mindfulness-based stress reduction for stress management in healthy people: a review and meta-analysis. **The journal of alternative and complementary medicine**, v. 15, n. 5, p. 593–600, 2009.

COHEN, S. **Cohen**, 1983.

COUR, P.; PETERSEN, M. Effects of Mindfulness Meditation on Chronic Pain : A Randomized Controlled Trial. p. 641–652, 2015.

CRESWELL, J. D. et al. Brief mindfulness meditation training alters psychological and neuroendocrine responses to social evaluative stress. **Psychoneuroendocrinology**, v. 44, p. 1–12, 2014.

DAUBENMIER, J. et al. It’s not what you think, it’s how you relate to it: Dispositional mindfulness moderates the relationship between psychological distress and the cortisol awakening response. **Psychoneuroendocrinology**, v. 48, p. 11–18, 2014.

DAVIS, L.; SETH KURZBAN. Mindfulness-Based Treatment for People with Severe Mental Illness: A Literature Review. **American Journal of Psychiatric Rehabilitation**, v. 15, n. 2, p. 202–232, 2012.

DECKRO, G. R. et al. The evaluation of a mind/body intervention to reduce psychological distress and perceived stress in college students. **Journal of American college health : J of ACH**, v. 50, n. 6, p. 281–7, 24 maio 2002.

DELORME, A.; MAKEIG, S. EEGLAB: An open source toolbox for analysis of single-trial EEG dynamics including independent component analysis. **Journal of Neuroscience Methods**, v. 134, n. 1, p. 9–21, 2004.

DIAS, J. C. R. et al. Perceived Stress Scale Applied to College Students: Validation Study. **Psychology, Community & Health**, v. 4, n. 1, p. 1–13, 2015.

DIAZ, B. A. et al. The Amsterdam Resting-State Questionnaire reveals multiple phenotypes of resting-state cognition. **Frontiers in human neuroscience**, v. 7, n. August, p. 446, 2013.

ESCH, T. et al. Mind/body techniques for physiological and psychological stress reduction: stress management via Tai Chi training - a pilot study. **Medical science monitor : international medical journal of experimental and clinical research**, v. 13, n. 11, p. CR488-R497, 2007.

FAN, Y. et al. Short Term Integrative Meditation Improves Resting Alpha Activity and Stroop Performance. **Applied Psychophysiology Biofeedback**, v. 39, n. 3–4, p. 213–217, 2014.

FARB, N. et al. Interoception, contemplative practice, and health. **Frontiers in Psychology**, v. 6, n. June, p. 763, 2015.

FARB, N. A S.; SEGAL, Z. V.; ANDERSON, A. K. Mindfulness meditation training alters cortical representations of interoceptive attention. **Social Cognitive and Affective Neuroscience**, v. 8, n. 1, p. 15–26, 2013.

GALINHA, I. C.; PAIS-RIBEIRO, J. L. Contribuição para o estudo da versão portuguesa da Positive and Negative Affect Schedule (PANAS): II – Estudo psicométrico. **Análise Psicológica**, v. 2, n. XXIII, p. 219–227, 2005.

GODSEY, J. The role of mindfulness based interventions in the treatment of obesity and eating disorders: an integrative review. **Complementary Therapies in Medicine**, v. 21, n. 4, p. 430–439, ago. 2013.

GOLDSTEIN, D. S. Catecholamines and stress. **Endocrine Regulations**, v. 37, n. 2, p. 69–80, 2003.

HASENKAMP, W.; BARSALOU, L. W. Effects of Meditation Experience on Functional Connectivity of Distributed Brain Networks. **Frontiers in Human Neuroscience**, v. 6, n. March, p. 1–14, 2012.

HAUS, E. Chronobiology in the endocrine system. **Advanced Drug Delivery Reviews**, v. 59, n. 9–10, p. 985–1014, 2007.

HOFMANN, S. G. et al. The effect of mindfulness-based therapy on anxiety and depression: A meta-analytic review. **Journal of consulting and clinical psychology**, v. 78, n. 2, p. 169–83, abr. 2010.

HOLZEL, B. K. et al. How Does Mindfulness Meditation Work? Proposing Mechanisms of Action From a Conceptual and Neural Perspective. **Perspectives on Psychological Science**, v. 6, n. 6, p. 537–559, 2011.

HÖLZEL, B. K. et al. Differential engagement of anterior cingulate and adjacent medial frontal cortex in adept meditators and non-meditators. **Neuroscience Letters**, v. 421, n. 1, p. 16–21, 2007.

HORCH, H. W. et al. Destabilization of cortical dendrites and spines by BDNF. **Neuron**, v. 23, n. 2, p. 353–364, 1999.

HUANG, H. PING et al. A meta-analysis of the benefits of mindfulness-based stress reduction (MBSR) on psychological function among breast cancer (BC) survivors. **Breast Cancer**, n. 12, 2015.

JHA, A. P.; KROMPINGER, J.; BAIME, M. J. Mindfulness training modifies subsystems of attention. **Cognitive, affective & behavioral neuroscience**, v. 7, n. 2, p. 109–19, jun. 2007.

KABAT-ZINN, J. **Full Catastrophe Living: Using the Wisdom of Your Body and Mind to Face Stress, Pain, and Illness**. [s.l.] Delta Trade Paperbacks, 1990.

KABAT-ZINN, J.; LIPWORTH, L.; BURNEY, R. The clinical use of mindfulness meditation for the self-regulation of chronic pain. **Journal of behavioral medicine**, v. 8, n. 2, p. 163–90, jun. 1985.

KANG, D. H. et al. The effect of meditation on brain structure: Cortical thickness mapping and diffusion tensor imaging. **Social Cognitive and Affective Neuroscience**, v. 8, n. 1, p. 27–33, 2013.

KILPATRICK, L. A. et al. Impact of mindfulness-based stress reduction training on intrinsic brain connectivity. **NeuroImage**, v. 56, n. 1, p. 290–298, 2011.

KOCH, A.; POLLATOS, O. Cardiac sensitivity in children: Sex differences and its relationship to parameters of emotional processing. **Psychophysiology**, v. 51, n. 9, p. 932–941, 2014.

KOCOVSKI, N. L. et al. Mindfulness and Acceptance-based Group Therapy and traditional Cognitive Behavioral Group Therapy for Social Anxiety Disorder: Mechanisms of Change. **Behaviour Research and Therapy**, v. 70, p. 11–22, 2015.

KOLOTYLOVA, T. et al. Entwicklung des mannheimer multikomponenten-stress-test (MMST). **PPmP Psychotherapie Psychosomatik Medizinische Psychologie**, v. 60, n. 2, p. 64–72, 2010.

KOPIN, I. J. Definitions of stress and sympathetic neuronal responses. **Annals of the New York Academy of Sciences**, v. 771, p. 19–30, 1995.

KURTH, F. et al. Brain Gray Matter Changes Associated with Mindfulness Meditation in Older Adults: An Exploratory Pilot Study using Voxel-based Morphometry. **Neuro : open journal**, v. 1, n. 1, p. 23–26, 2015.

KUYKEN, W. et al. Effectiveness and cost-effectiveness of mindfulness-based cognitive therapy compared with maintenance antidepressant treatment in the prevention of depressive relapse or recurrence (PREVENT): a randomised controlled trial. **The Lancet**, v. 386, n. 9988, p. 63–73, 2015.

LAZAR, S. W. et al. Meditation experience is associated with increased cortical thickness. **Neuroreport**, v. 16, n. 17, p. 1893–7, 28 nov. 2005.

LICINIO, J.; WONG, M.-L. Brain-derived neurotrophic factor (BDNF) in stress and affective disorders. **Molecular Psychiatry**, v. 7, n. 6, p. 519–519, 2002.

LIOTTI, M. et al. Brain responses associated with consciousness of breathlessness (air hunger). **Proceedings of the National Academy of Sciences**, v. 98, n. 4, p. 2035–2040, 2001.

MALINOWSKI, P. Neural mechanisms of attentional control in mindfulness meditation. **Frontiers in Neuroscience**, v. 7, n. 7 FEB, p. 1–11, 2013.

MARCHAND, W. R. Mindfulness-based stress reduction, mindfulness-based cognitive therapy, and Zen meditation for depression, anxiety, pain, and psychological distress. **Journal of Psychiatric Practice**, v. 18, n. 4, p. 233–252, jul. 2012.

MCEWEN, B. S.; WINGFIELD, J. C. What is in a name? Integrating homeostasis, allostasis and stress. **Hormones and behavior**, v. 57, n. 2, p. 105–11, fev. 2010.

MEHLING, W. E. et al. The Multidimensional Assessment of Interoceptive Awareness (MAIA). **PLoS ONE**, v. 7, n. 11, 2012.

MEHLING, W. E. Multidimensional Assessment of Interoceptive Awareness. v. 1, n. 415, 2012.

MURAKAMI, S. et al. Chronic stress, as well as acute stress, reduces BDNF mRNA expression in the rat hippocampus but less robustly. **Neuroscience Research**, v. 53, n. 2, p. 129–139, 2005.

O’LEARY, K.; O’NEILL, S.; DOCKRAY, S. A systematic review of the effects of mindfulness interventions on cortisol. **Journal of health psychology**, n. March 2016, p. 1359105315569095-, 2015.

O’REILLY, G. et al. Minfulness-Based Interventions for Obesity-Related Eating Behaviors: A Literature Review. **Obes Rev**, v. 15, n. 6, p. 453–461, 2014.

REINHARDT, T. et al. Salivary cortisol, heart rate, electrodermal activity and subjective stress responses to the Mannheim Multicomponent Stress Test (MMST). **Psychiatry Research**, v. 198, n. 1, p. 106–111, 2012.

RICCIO, C. A. et al. The continuous performance test: A window on the neural substrates for attention? **Archives of Clinical Neuropsychology**, v. 17, n. 3, p. 235–272, 2002.

ROBERTSON, I. H. et al. “Oops!”: Performance correlates of everyday attentional failures in traumatic brain injured and normal subjects. **Neuropsychologia**, v. 35, n. 6, p. 747–758, 1997.

SANDERS, A. F. Towards a Model of Stress and Human. **Acta Psychologica**, v. 53, p. 61–97, 1983.

SAPOLSKY, R. M.; ROMERO, L. M.; MUNCK, A. U. How Do Glucocorticoids Influence Stress Responses ? Preparative Actions *. **Endocrine Reviews**, v. 21, n. April, p. 55–89, 2000.

SCHANDRY, R. Heart Beat Perception and Emotional Experience. **Psychophysiology**, v. 18, n. 4, p. 483–488, 1981.

SELMAOUI, B.; TOUITOU, Y. Reproducibility of the circadian rhythms of serum cortisol and melatonin in healthy subjects: A study of three different 24-h cycles over six weeks. **Life Sciences**, v. 73, n. 26, p. 3339–3349, 2003.

STRATAKIS, C. A.; CHROUSOS, G. P. Neuroendocrinology and pathophysiology of the stress system. **Annals of the New York Academy of Sciences**, v. 771, p. 1–18, 1995.

TAKAHASHI, M. et al. Abnormal expression of brain-derived neurotrophic factor and its receptor in the corticolimbic system of schizophrenic patients. **Molecular psychiatry**, v. 5, n. 3, p. 293–300, 2000.

TANAY, G.; BERNSTEIN, A. State Mindfulness Scale (SMS): development and initial validation. **Psychological assessment**, v. 25, n. 4, p. 1286–99, 2013.

TANG, Y.-Y. et al. Short-term meditation training improves attention and self-regulation. **Proceedings of the National Academy of Sciences of the United States of America**, v. 104, n. 43, p. 17152–17156, 2007.

TANG, Y.-Y.; HÖLZEL, B. K.; POSNER, M. I. Traits and states in mindfulness meditation. **Nature Reviews Neuroscience**, v. 17, n. DECEMBER, p. 59–59, 2015a.

TANG, Y.-Y.; HÖLZEL, B. K.; POSNER, M. I. The neuroscience of mindfulness meditation. **Nature Reviews Neuroscience**, v. 16, n. 4, p. 1–13, 2015b.

TSIGOS, C.; CHROUSOS, G. P. Hypothalamic-pituitary-adrenal axis, neuroendocrine factors and stress. **Journal of Psychosomatic Research**, v. 53, n. 4, p. 865–871, 2002.

UEBELACKER, L. A.; BROUGHTON, M. K. Yoga for Depression and Anxiety: A Review of Published Research and Implications for Healthcare Providers. **Rhode Island medical journal (2013)**, v. 99, n. 3, p. 20–2, jan. 2016.

ULRICH-LAI, Y. M.; HERMAN, J. P. Neural regulation of endocrine and autonomic stress responses. **Nature reviews. Neuroscience**, v. 10, n. 6, p. 397–409, 2009.

VALDEZ, P. et al. Circadian rhythms in components of attention. **Biological Rhythm Research**, v. 36, n. 1–2, p. 57–65, 2005.

WANG, F. et al. The effects of tai chi on depression, anxiety, and psychological well-being: a systematic review and meta-analysis. **International journal of behavioral medicine**, v. 21, n. 4, p. 605–17, ago. 2014.

WATSON, D.; CLARK, L. A; TELLEGEN, A. Development and validation of brief measures of positive and negative affect: the PANAS scales. **Journal of personality and social psychology**, v. 54, n. 6, p. 1063–1070, 1988.

WÜRTZEN, H. et al. Effect of mindfulness-based stress reduction on somatic symptoms, distress, mindfulness and spiritual wellbeing in women with breast cancer: Results of a randomized controlled trial. **Acta Oncologica**, v. 54, n. August 2014, p. 1–8, 2015.

ZHOU, R.; LIU, L. Eight-Week Mindfulness Training Enhances Left Frontal EEG Asymmetry During Emotional Challenge: a Randomized Controlled Trial. **Mindfulness**, p. 1–9, 2016.

ZYLOWSKA DEBORAH ACKERMAN MAY H YANG JULIE L FUTRELL NANCY L HORTON T SIGI HALE, L. L.; PATAKI, C.; SMALLEY, S. L. Mindfulness Meditation Training in Adults and Adolescents With ADHD A Feasibility Study. **Journal of Attention Disorders**, v. 11, n. 6, p. 737–746, 2008.

**Audio Transcript- Mindfulness-based exercise (30min)**

Thank you for being part of our research.

Find a relaxed, comfortable position in which you can spend the next minutes.

With your feet touching the floor, the spine erected but not rigid.

Perhaps you can notice the sounds, the temperature, the touch of your clothes

in your skin.

Maybe you can notice all your body, doing a quickly body scan by paying

attention to your feet, your hips, the touch of your hips with the chair.

With your eyes closed, become curious about your body and be aware of the

sensations of your breath

Whatever thoughts come and go through your mind, simply observe and then

allow yourself to notice your breathing.

Notice your belly, and the movements it makes when you inhale and exhale.

Be aware of your chest and the movements it makes when you breathe.

Be aware of your body breathing at this moment.

You don’t need to control your breath. Just follow your own rhythm.

If you notice that you got lost in your own thoughts, gently return your attention

to your body, to the sensations of your breath.

Be aware of the movements your body makes when you inhale and exhale.

Return your awareness to your own body. Notice if there is any areas of

tightness or tension. See if you can allow them to soften when you breathe.

Notice how your body reacts to your breathing, how do you feel

when you breathe with awareness and let go any thoughts that may come to your

experience.

If you notice that your mind started to wander, gently and nonjudgmentally

redirect your attention right back to your breathing.

Don’t feel guilty or judge yourself for get lost, this is completely normal. Just act with acceptance and redirect your attention to your breath.

Perhaps you can pay attention to your nostrils, welcoming the sensations that

are present when you breath. Maybe you can notice the air coming in and out of

your body.

Whatever thoughts come and go through your mind at this point, just let them

go and gently redirect your attention right back to the breathing.

Maybe you can notice if there is any difference between the temperature when

you inhale and exhale.

Simple allow your body breath and observe your experience unfolding moment

by moment

Let your attention gently come to rest on your breathing.

Notice the physical sensations of breathing, when you inhale and exhale.

Remember you don’t need to judge yourself if your mind starts wandering.

If that happens, gently, non-judgmentally, bring back your attention to your

breath.

Be aware of your body. Notice how you feel at this moment.

Notice if there is any areas of tightness or tension, and breathe, relaxing.

Notice the physical sensations of breathing. Maybe you can relax your attention

and breath mindfully for next moments.

Feel all your body and allow yourself to be here, now.
