# Supplementary material for: The impact of a brief mindfulness training on interoception: A randomized controlled trial
Source: PLoS One. 2022 Sep 7;17(9):e0273864. doi: 10.1371/journal.pone.0273864 (PMC9451078; doi:10.1371/journal.pone.0273864)
Supplement: S2 Protocol — (DOCX) [file pone.0273864.s006.docx]

UNIVERSIDADE FEDERAL DO RIO GRANDE DO NORTE

PROGRAMA DE PÓS-GRADUAÇÃO EM NEUROCIÊNCIAS

INSTITUTO DO CÉREBRO

**Título da pesquisa:** IMPACTO DE PRÁTICAS BASEADAS EM MINDFULNESS SOBRE A RESPOSTA AO ESTRESSE, REGULAÇÃO EMOCIONAL, HABILIDADE INTEROCEPTIVA E ATENCIONAL EM INDIVÍDUOS SAUDÁVEIS

**Número de aprovação:** 1.761.383

**CAAE:** 55193416.4.0000.5537

**Pesquisador Principal:**

Maria Bernardete Cordeiro de Sousa

**Pesquisadores associados:**

Geissy Lainny de Lima Araújo

Geovan Menezes de Sousa Junior

Thatiane M. A. S. Mendes

Marcelo Demarzo

Norman Farb

Draulio B Araujo

**Instituição proponente**

Instituto do Cérebro

Universidade Federal do Rio Grande do norte- UFRN

Av. Senador Salgado Filho, 3.000, Campus Universitário, Lagoa Nova
CEP: 59078-900, Caixa Postal 1524, Natal/RN

**Financiamento**

CNPq 484972/2013‑8 e 306051/2017-6.

**Resumo**

Atualmente, adultos jovens vêm sendo expostos a sobrecarga de trabalho e estudo e, por isso, muitas vezes acabam desenvolvendo quadros de doenças relacionadas ao estresse. Os programas baseados em *mindfulness* para redução do estresse (MBSR) vêm sendo amplamente realizados nos mais diversos contextos, sendo tratados como possíveis terapias para quadros de depressão, ansiedade, dor crônica e estresse crônico. Originalmente criado como um programa de oito semanas, agora ele vem sendo adaptado para uma intervenção breve visando uma maior aderência às atividades e ao programa em si. A relação das práticas com uma melhor regulação emocional e aumento da capacidade de atenção em atividades da vida diária vem sendo demonstrada. No entanto, o componente interoceptivo que faz parte diretamente das práticas baseadas em *mindfulness* e está relacionado ao bem-estar e maior capacidade de autorregulação, não é bem compreendido. Nesse sentido, o presente estudo busca entender como se dá a interação entre os benefícios trazidos pela prática breve ou longa baseadas em *mindfulness* do ponto de vista de atividade cerebral, regulação emocional, habilidade atencional e interoceptiva em indivíduos saudáveis submetidos a um estímulo estressor. Para isso, serão avaliados níveis de cortisol sanguíneo e salivar e aplicação de testes cognitivos direcionados a atenção e regulação emocional, além de instrumentos psicométricos que visam avaliar a interocepção, níveis de ansiedade, depressão, estresse e *mindfulness* como traço da personalidade ou como estado de consciência.

**1. Introdução**

Nas últimas décadas a literatura tem apresentado a utilização de novas abordagens com foco integrativo mente-corpo com o intuito de prevenir ou tratar quadros de enfermidades que têm aumentado com o estilo de vida adotado nas sociedades atuais como ansiedade, depressão e estresse (ESCH et al., 2007; MARCHAND, 2012; UEBELACKER; BROUGHTON, 2016; WANG et al., 2014). O estresse pode ser caracterizado como qualquer perturbação a homeostase do organismo proporcionada por estímulos exteroceptivos (ex: cheiro de um predador, informação visual) ou estímulos interoceptivos (volume sanguíneo, osmolaridade) (ULRICH-LAI; HERMAN, 2009). No âmbito social e psicológico, um levantamento realizado nos Estados Unidos demonstrou que o estresse apresenta como principais fatores desencadeadores a situação econômica, emprego, família e estado de saúde (APA, 2014). O quadro de estresse se manifesta em indivíduos de todas as idades, especialmente em jovens adultos que, muitas vezes, são submetidos a rotinas de estudo e trabalho, paralelamente, levando a uma sobrecarga biopsicossocial.

A síndrome de adaptação geral ou resposta ao estresse foi primeiramente descrita por Hans Selye em 1936 (Selye, 1936) e tem como objetivo manter a integridade fisiológica do organismo. Portanto, essa resposta é extremamente necessária à sobrevivência desde que se mantenha em níveis ótimos, sendo sua ausência ou sua exacerbação responsáveis por desordens fisiológicas importantes e respostas inapropriadas a uma demanda específica (MCEWEN; WINGFIELD, 2010). É mediada principalmente pelo eixo Hipotálamo-Pituitária-Adrenal (HPA) através de uma cascata de eventos celulares que levam a liberação de hormônios na corrente sanguínea. Um dos principais marcadores da reatividade do eixo HPA ao estresse é o hormônio cortisol, que tem sua liberação regulada pelo relógio biológico em um ritmo circadiano e também através de situações estressoras à homeostase do organismo (HAUS, 2007; SELMAOUI; TOUITOU, 2003). Essa reatividade pode ser mensurada através de várias técnicas, entre as quais figuram a investigação a partir do plasma sanguíneo, urina e saliva. A partir de um estímulo estressor, há a ativação de neurônios localizados no núcleo paraventricular (PVN) do tálamo que produzem e secretam o fator de liberação do hormônio corticotrófico no sistema porta-hipofisário. Posteriormente, há a produção e liberação, a partir de adenohipófise, do hormônio adenocorticotrófico (ACTH) que, através da corrente sanguínea chega até o córtex da glândula adrenal, promovendo a produção e liberação de glicocorticoides, como o cortisol. O cortisol atua por feedback negativo sobre o hipotálamo e a hipófise de modo que, ao atingir altas concentrações no sangue, inibe a liberação de fatores que propiciam a sua produção (SANDERS, 1983; SAPOLSKY; ROMERO; MUNCK, 2000; STRATAKIS; CHROUSOS, 1995; TSIGOS; CHROUSOS, 2002; ULRICH-LAI; HERMAN, 2009). Além da ativação do eixo HPA, há uma importante regulação do sistema nervoso autônomo (SNA) durante a resposta ao estresse, como, por exemplo, a alteração da atividade cardíaca, mediada principalmente pela liberação de catecolaminas, que atuam também sobre o núcleo PVN do tálamo e sobre regiões límbicas (GOLDSTEIN, 2003; KOPIN, 1995; ULRICH-LAI; HERMAN, 2009). Além disso, neuromoduladores como o BDNF (brain derived neurotrophic fator- Fator neurotrófico derivado do cérebro) e a expressão de seus receptores também são afetados em caso de estresse agudo ou crônico, efeito observado em estudos com roedores e com humanos (LICINIO; WONG, 2002; MURAKAMI et al., 2005; TAKAHASHI et al., 2000). O BDNF está principalmente relacionado com plasticidade sináptica e neurogênese, e alterações em sua liberação ou na expressão dos seus receptores pode causar déficits em processos básicos importantes como a aquisição de uma nova memória ou desenvolvimento de transtornos psiquiátricos (ALLEVA; SANTUCCI, 2001; BERTON et al., 2006; HORCH et al., 1999; MURAKAMI et al., 2005; TAKAHASHI et al., 2000). A resposta cerebral a estímulos estressores (estímulo não físico ou psicogênico) baseia-se em experiência prévia a partir da ativação de circuitos neurais anteriormente estabelecidos ou em programas inatos do organismo. Essas reações geralmente são processadas no prosencéfalo, envolvendo o sistema límbico e podem ocorrer em antecipação ou em resposta a um estímulo estressor, para revisão ver (ULRICH-LAI; HERMAN, 2009).

Programas baseados em *mindfulness*

A busca por meios de reduzir o impacto da rotina e pressão social sobre a saúde levou ao desenvolvimento de alternativas preventivas e terapêuticas para quadros de estresse. Uma delas é o uso de práticas meditativas sistematizadas em oito semanas conhecido como programa de práticas baseadas em *mindfulness* para redução do estresse (MBSR- *Mindfulness* Based Stress Reduction)

Quando traduzida para o português, a palavra “*mindfulness*” pode ser encontrada como atenção plena, consciência plena, atenção consciente. *Mindfulness* é descrito por alguns autores como um estado consciente e atento ao que acontece no momento presente (KABAT-ZINN, 1990). O *mindfulness* pode ser tratado como um traço de personalidade, como um estado mental ou também pode estar relacionado a um contexto de programas que utilizam práticas envolvendo o treinamento da atenção (TANG; HÖLZEL; POSNER, 2015a).

As práticas meditativas baseadas em *mindfulness* tiveram seu início em um contexto clínico na década de 70 através do desenvolvimento de um programa para manejo do estresse e da dor crônica (KABAT-ZINN, 1990; KABAT-ZINN; LIPWORTH; BURNEY, 1985). Atualmente, existem diversas modalidades de programas terapêuticos baseados em *mindfulness* voltados a patologias ou grupos específicos. Dentre as mais conhecidas, figuram o MBSR, o MBCT (*Mindfulness* based cognitive therapy) e o MBRP (*Mindfulness* based relapse prevention). De maneira geral, as práticas baseadas em *mindfulness* para redução do estresse compõem um programa de treinamento bem estabelecido ao longo de 8 (oito) semanas. Nesse programa, desenvolvem-se atividades que usam como ferramentas as práticas meditativas voltadas a refinar a atenção e a autoconsciência de quem as pratica (KABAT-ZINN, 1990). O que se cultiva nessa prática é um estado atento ao momento presente, com abertura, curiosidade e sem julgamento, onde o foco atencional é direcionado para a observação da experiência dos pensamentos, nas sensações corporais e nas emoções (HOLZEL et al., 2011; KABAT-ZINN, 1990).

Os efeitos desse treinamento vêm sendo demonstrados no tratamento de diferentes enfermidades com respostas em níveis estruturais e funcionais do sistema nervoso central (SNC) e, consequentemente, na expressão do comportamento do indivíduo. Desordens como ansiedade (KOCOVSKI et al., 2015), depressão (HOFMANN et al., 2010) , abuso de substâncias (BOWEN et al., 2007, 2014) e transtorno alimentar (GODSEY, 2013; O’REILLY et al., 2014) estão entre as mais estudadas no contexto da aplicação de *mindfulness* como terapia. Além disso, a reabilitação após câncer de mama (HUANG et al., 2015; WÜRTZEN et al., 2015) e casos de dor crônica (BAWA et al., 2015; COUR; PETERSEN, 2015; KABAT-ZINN; LIPWORTH; BURNEY, 1985) também caracterizam uma grande parte dos estudos realizados nos últimos 30 anos.

Com a diversidade de trabalhos encontrados na literatura acerca da aplicação dos programas baseados em *mindfulness*, observa-se três linhas principais onde concentram-se os estudos: treinamento da atenção, beneficiando pessoas com transtorno do déficit de atenção e hiperatividade ou quadro semelhante (ZYLOWSKA DEBORAH ACKERMAN MAY H YANG JULIE L FUTRELL NANCY L HORTON T SIGI HALE; PATAKI; SMALLEY, 2008), melhor regulação emocional a partir do reconhecimento de modos automáticos de pensamentos ou sentimentos em pessoas com depressão (KUYKEN et al., 2015), e o desenvolvimento de um autoconhecimento e melhora da resiliência diante de situações estressoras em pessoas com alguma desordem a nível mental (DAVIS; SETH KURZBAN, 2012).

O MBSR (voltado especificamente para redução de estresse) tem sido amplamente estudado em indivíduos saudáveis ou com alguma patologia e seu efeito vem sendo demonstrado sobre o eixo neuroendócrino com intervenções de curto e de longo prazo (BROWN; WEINSTEIN; CRESWELL, 2012; CHIESA; SERRETTI, 2009; CRESWELL et al., 2014; DECKRO et al., 2002; O’LEARY; O’NEILL; DOCKRAY, 2015).

Creswell e colaboradores em 2014 demonstraram que uma prática de 3 dias de exercícios do MBSR melhoraram a percepção de estresse dos participantes quando comparados a um grupo controle. Nesse estudo, os participantes foram submetidos ao teste de estresse social (TSST- Trier Stress Social Test) após as intervenções e foi verificado que pessoas que tem um traço de *mindfulness* mais acentuado antes da intervenção tem menor reatividade do eixo neuroendócrino diante de um estresse agudo. Porém, comparativamente ao grupo controle, o grupo submetido a prática de *mindfulness* teve, em geral, um aumento no nível de cortisol salivar (CRESWELL et al., 2014). De fato, o traço de *mindfulness* parece influenciar nos níveis de cortisol e na relação do indivíduo com emoções negativas, sendo aqueles com menor traço mais propícios a disfunções emocionais e os com maior traço apresentam melhor adaptação frente a um estímulo estressor (BROWN; WEINSTEIN; CRESWELL, 2012; DAUBENMIER et al., 2014).

Já em um estudo realizado utilizando um programa de 6 semanas com práticas baseadas em *mindfulness* em estudantes, demonstrou menores níveis de estresse percebido, sintomas de ansiedade e depressão pós treinamento (DECKRO et al., 2002).

Em uma revisão sistemática acerca da relação da prática de *mindfulness* com níveis de cortisol, os autores relatam a impossibilidade de uma conclusão comum acerca dos resultados apresentados. Isso ocorre devido a falta de randomização ou a problemas metodológicos encontrados nos estudos. (O’LEARY; O’NEILL; DOCKRAY, 2015).

*Mindfulness*, regulação emocional, atencional e habilidade interoceptiva

Recentemente alguns estudos demonstraram alterações estruturais e funcionais observadas no cérebro de meditadores experientes (HASENKAMP; BARSALOU, 2012; HÖLZEL et al., 2007; KANG et al., 2013; KILPATRICK et al., 2011; KURTH et al., 2015; LAZAR et al., 2005). Em linhas gerais, áreas cerebrais que apresentam atividade diferenciada em praticantes de atividades meditativas consistem em regiões corticais e subcorticais envolvidas com o sistema límbico. São elas o córtex pré-frontal, região relacionada à meta-cognição, ao planejamento e ao julgamento; hipocampo, área relacionada com a processamento de memórias; córtex cingulado anterior e orbitofrontal, áreas relacionadas com a percepção do “self”, regulação atencional e emocional; ínsula, região relacionada com interocepção; amígdala, região relacionada com processamento emocional, além de áreas envolvidas com a comunicação inter-hemisférica como o corpo caloso e o fascículo longitudinal superior para revisão ver (TANG; HÖLZEL; POSNER, 2015b). Interessantemente, regiões do sistema límbico parecem estar relacionadas com a regulação do núcleo paraventricular do hipotálamo, local importante que compõe o eixo HPA. Dessa maneira, alterações de ativação de regiões límbicas podem levar a diferenças fisiológicas na resposta a algum evento estressor (ULRICH-LAI; HERMAN, 2009).

Um dos principais componentes das atividades baseadas em *mindfulness* é a regulação da atenção (BISHOP et al., 2004). A partir da execução dessas práticas, têm sido observadas alterações nos subcomponentes do sistema atencional como a orientação, o alerta e o monitoramento de conflitos, em diferentes contextos (MALINOWSKI, 2013; TANG; HÖLZEL; POSNER, 2015b).

As práticas de *mindfulness* envolvem atenção focada, quando utiliza um foco fixo durante sua execução e é presente principalmente no início das práticas, e monitoramento aberto, quando o indivíduo desenvolve a capacidade de mover naturalmente sua atenção entre os elementos da experiência (MALINOWSKI, 2013). De maneira geral, os estudos demonstram uma melhor performance em testes atencionais em meditadores experientes e isso é correlacionado com áreas corticais diretamente envolvidas no processo de atenção (MALINOWSKI, 2013; TANG; HÖLZEL; POSNER, 2015b). Jha e colaboradores em 2007 demonstraram melhor performance no teste de rede de atenção (Attentional network test- ANT) de indivíduos submetidos a um MBSR de 8 semanas e indivíduos que participaram de um retiro de um mês e realizaram práticas de atenção focada e monitoramento aberto, corroborando com outros estudos contemporâneos a esse (JHA; KROMPINGER; BAIME, 2007; TANG et al., 2007).

Ainda em relação ao componente atencional, com o uso da eletroencefalografia (EEG), Fan e colaboradores demonstraram um aumento no potencial da onda alfa em regiões fronto-parietais durante um teste de atenção após a prática de exercícios baseados em *mindfulness*. O aumento do potencial dessas ondas, nessa região, parece estar associado a uma melhor performance cognitiva, refletida através de um menor tempo de reação ao estímulo além de menor quantidade de erros ao longo da tarefa (FAN et al., 2014). Além disso, a redução da assimetria de alfa frontal também tem sido reportada após um programa de 8 semanas de *mindfulness*. Este resultado parece relacionar-se com uma melhor capacidade de regulação emocional envolvendo maior bem-estar e menor reatividade a situações emocionalmente negativas (ZHOU; LIU, 2016).

A interocepção ou habilidade interoceptiva pode ser descrita como a capacidade do indivíduo tomar ciência dos sinais originados a partir do próprio corpo (FARB et al., 2015). Essa capacidade tem sido relacionada a um maior bem-estar e a uma melhor autorregulação, tendo em vista que ela é necessária para a percepção de eventos internos importantes para a sobrevivência (FARB et al., 2015; LIOTTI et al., 2001). Em um estudo recente, foi observado uma mudança interessante no autorrelato de aspectos pessoais como a atenção ao corpo após 3 meses de práticas contemplativas (BORNEMANN et al., 2015). Juntamente a mudanças no autorrelato, foi mensurada a mudança de atenção interoceptiva através de um instrumento desenvolvido para esse fim denominado Avaliação multidimensional da consciência interoceptiva (MEHLING et al., 2012).

Farb e colaboradores em 2013 demonstraram que a prática de *mindfulness* leva a uma maior atividade cortical de regiões relacionadas a interocepção, como a porção anterior da ínsula (FARB; SEGAL; ANDERSON, 2013). Levando em conta que grande parte das práticas de *mindfulness* utilizam a atenção voltada a eventos internos ou externos, o componente interoceptivo como benefício das práticas deve ser levado em consideração.

No entanto, não encontra-se na literatura nenhum estudo que demonstre os efeitos de uma intervenção baseada em mindfulness sobre a atividade cortical durante tarefas cognitivas e interoceptivas diante de um estímulo estressor.

**2. Objetivos**

Geral:

- Avaliar o impacto de um breve treinamento baseado em *mindfulness* na resposta ao estresse e em medidas de interocepção e bem-estar em indivíduos saudáveis;

Específicos:

- Verificar a relação entre traço de *mindfulness* e reatividade do eixo hipotálamo-hipófise-adrenal;
- Analisar a resposta neuroendócrina a um evento de estresse social após uma intervenção breve baseada em *mindfulness*;
- Avaliar o impacto da prática de *mindfulness* (3 dias) sobre o sistema atencional, sistema nervoso autônomo e habilidade interoceptiva.
- Verificar a influência da prática sobre o autorrelato de quadros de estresse, ansiedade e afetividade.
- Avaliar atividade eletroencefalográfica dos participantes antes e após as intervenções propostas, durante um teste de atenção sustentada, um teste de interocepção e durante o teste de estresse.

**3. Materiais e Métodos**

**Participantes**

Serão recrutados 50 jovens adultos de ambos os sexos, com faixa etária entre 18-35 anos através de ampla divulgação no campus da Universidade Federal do Rio Grande do Norte- UFRN. O tamanho da amostra foi determinado com G * Power da família de testes F (RM-ANOVA com interação intra-entre), considerando um tamanho de efeito moderado a alto (f = 0,3) em α = 0,05 e 80% do poder, bem como 60% de correlação entre medidas repetidas e uma perda voluntários durante o estudo, sendo necessários pelo menos 40 participantes para atender a esses parâmetros.

Os participantes serão previamente entrevistados e responderão a uma série de questionários para verificar sua elegibilidade para o estudo. Ao serem elegidos para o estudo, serão randomizados em dois grupos propostos nesse estudo.

**Critérios de inclusão:**

- Ter entre 18 e 35 anos;
- Não possuir nenhum diagnóstico clínico psiquiátrico (esquizofrenia, depressão maior, transtorno bipolar, mania, transtorno de ansiedade social);
- Não fazer uso de medicamentos psicotrópicos, beta-bloqueadores ou anti-inflamatórios no momento do estudo;
- Não ter experiência prévia com práticas baseadas em *mindfulness*;
- Assinar o TCLE.

**Critérios de exclusão**

- Ausentar-se algum dia do protocolo de estudo (intervenção breve);
- Apresentar alguma doença infecciosa durante o estudo
- Índice de massa corporal (IMC) acima de 30.

**Intervenção breve**

Tendo como base estudos anteriores, será realizada uma intervenção breve durante 3 dias durante 30 minutos por dia, envolvendo práticas contidas no programa de *mindfulness* para redução do estresse. O exercício utilizado no estudo será uma prática de atenção focada nas sensações da respiração que será gravado por um profissional qualificado. O participante virá ao laboratório no período da manhã durante 3 dias consecutivos e deverá manter-se em uma sala destinada às práticas durante todo o procedimento. O grupo controle passará o mesmo tempo em uma sala destinada ao estudo e será submetido a uma tarefa de colorir figuras durante o mesmo tempo do grupo experimental. As etapas do estudo estão especificadas no esquema abaixo:

Figura 1: Linha temporal a aplicação das avaliações ao longo do estudo do grupo 1 e 2. Sendo EEG: Eletroencefalografia.

De modo esquemático, a avaliação com o EEG será realizada da seguinte maneira:

Figura 2: Esquema demonstrando as atividades a serem realizadas durante a aquisição de dados utilizando a eletroencefalografia (EEG).

**Indução de estresse e avaliação da regulação emocional**

O teste de estresse a ser utilizado é o Mannheim multicomponent stress test (MMST) (KOLOTYLOVA et al., 2010). O MMST é um teste de componentes múltiplos que envolve estímulo emocional, auditivo e aritmético durante 5 minutos. A resposta do eixo HPA e do SNA parece ser similar a obtida com o TSST (Trier Social Stress Test), o teste padrão utilizado para verificar resposta ao estresse em estudos laboratoriais. (REINHARDT et al., 2012). Nos primeiros 3 minutos do teste, o participante é apresentado a várias imagens com valência emocional positiva, negativa e neutra e é solicitado a categorizar a imagem nessas valências em uma escala de 1-7. Serão apresentadas 52 imagens no total. Após isso, um ruído branco que varia de 78-98dB é apresentado e o participante é solicitado a realizar um teste aritmético de acordo com o que aparece na tela do computador a sua frente. Esse teste é o PASAT (Paced Auditory Serial Addition Test) no qual o participante deve adicionar o último dígito demonstrado ao imediatamente anterior a ele, digitando a soma de ambos os dígitos no teclado a sua frente.

**Coletas e dosagens bioquímicas**

Eletroencefalografiam (EEG), Eletrocardiograma (ECG) e resposta galvânica da pele (GRS):

A eletroencefalografia será realizada com o aparelho da BrainAmp DC (Brain Products GmbH, Munique, Alemanha) de 64 canais, utilizando eletrodos ativos ActiCap (Brain Products GmbH, Munique, Alemanha). Os eletrodos de ECG e GSR serão conectados ao BrainAmp ExG (Brain Products GmbH, Munique, Alemanha). Os dados do EEG serão amalisados através do software Matlab utilizando a toolbox EEGLAB (DELORME; MAKEIG, 2004).

**Saliva:**

Amostras de saliva serão coletadas antes do início do teste de estresse e 15, 30 e 60 minutos após o seu fim. As amostras serão coletadas em dispositivo apropriado - Salivettes (Sarstedt, Germany) e posteriormente processadas, centrifugadas em 3000 RPM, durante 15 minutos e armazenadas a -30º C até posterior dosagem.

**Dosagem de cortisol salivar**

As dosagens de cortisol salivar serão realizadas a partir das amostras coletadas no dia 3 do protocolo. As mesmas serão feitas utilizando kits comerciais de dosagem da empresa DRG Instruments GmbH (Germany).

O princípio básico utilizado pelo kit é o do ELISA por competição, no qual uma microplaca é sensibilizada com anticorpos (IgG) monoclonais para cortisol. A leitura das amostras serão realizadas por espectrofotometria.

No dia 1 do protocolo o participante responderá aos questionários pré-intervenção e será coletada uma amostra de sangue para posterior análise de cortisol e BDNF.

**Coleta de sangue:**

Os participantes do estudo serão submetidas a uma punção venosa de 20 ml de sangue após jejum de 8 horas antes e após a intervenção proposta nesse projeto. Elas serão orientadas a terem uma noite tranquila de sono e comparecer ao local de coleta no horário combinado. A coleta de sangue será realizada no braço não dominante de cada participante, no período da manhã (entre 8:00 e 9:30 horas) após 45 minutos de repouso do participante. Amostras de sangue periférico serão colhidas sem anticoagulante (10 mL) para o estudo do perfil hematológico, lipídico, hepático e renal e do marcador endócrino (cortisol) e do BDNF.

**Dosagem do cortisol plasmático**

 Os níveis do cortisol serão determinados em duplicata pelo método imunoenzimático (ELISA). Os coeficientes de variação serão calculados e deverão ser menores do que 20%. O princípio básico utilizado pelo kit é o ensaio por competição, no qual uma microplaca é sensibilizada com anticorpos (IgG) monoclonais para cortisol. As amostras desconhecidas (dos participantes) competem com o cortisol ligado a uma enzima peroxidase pelos anticorpos citados. Após um período de incubação, os componentes não ligados são lavados. Um substrato (tetrametilbenzidina) é adicionado e oxidará a enzima ligada ao cortisol produzindo uma mudança de coloração. Com a adição de uma solução de parada, ocorre outra mudança de coloração e em seguida será feita a leitura espectrofotométrica utilizando filtro com comprimento de onda adequado. Vale salientar que por esse tipo de ELISA, quanto maior a densidade óptica, menor a quantidade de hormônio presente na amostra, sendo o inverso verdadeiro.

**Dosagem do BDNF**

Alíquotas de 2mL de plasma serão também separadas após a coleta de sangue, para análise do BDNF, através da técnica de ensaio imunoenzimático (ELISA), com utilização de kits comerciais.

**Avaliação do Sistema Nervoso Autônomo**

A mensuração da modulação do Sistema Nervoso Autonômico (SNA) sobre o coração será realizada com a técnica de Variabilidade da frequência cardíaca (VFC) que utiliza os intervalos R-R do eletrocardiograma (ECG) captados e registrados durante uma hora com os voluntários em vigília com eletrodos conectados ao BrainAmp ExG (Brain Products GmbH, Munique, Alemanha).

Serão empregada medidas de domínio de tempo e de frequência (HF (High frequency), LF (Low frequency), VLF (Very low frequency) e Razão (LF/HF) para a representação da atividade vagal cardíaca.

**Avaliação interoceptiva**

Além da aplicação da escala destinada a avaliação interoceptiva (MAIA) anteriormente descrita, faremos uma avaliação objetiva baseada em estudos anteriores (KOCH; POLLATOS, 2014; SCHANDRY, 1981).

O teste de contagem dos batimentos cardíacos será utilizado para verificar a acurácia cardíaca que reflete o nível de sensibilidade interoceptiva dos voluntários. Este teste é bastante utilizado na literatura e a interocepção cardíaca é representativa da interocepção corporal. O teste consiste da contagem silenciosa sem manuseio tátil do número dos próprios batimentos cardíacos percebidos durante três períodos de tempo 25s, 35s e 45s, que são combinados de forma aleatória, intercalados por 30s (SCHANDRY, 1981). Posteriormente, o número de batimentos percebido é comparado com o número de batimentos real, resultando o nível de acurácia cardíaca. O protocolo de contagem com os três períodos de tempo intercalados por 30s será repetido por três vezes, e será considerada a média dos dois valores de acurácia cardíaca mais próximos. A repetição deste procedimento em dias diferentes será realizada para assegurar a reprodutibilidade do nível de acurácia interoceptiva dos voluntários. Os voluntários serão classificados com alta e baixa sensibilidade interoceptiva a partir do critério de sensibilidade acima ou abaixo de 75% de acurácia, respectivamente.

**Atenção Sustentada**

Para verificar o componente atencional, realizaremos um teste de atenção sustentada. O teste será sempre no mesmo horário para evitar influências do ritmo circadiano (RICCIO et al., 2002; VALDEZ et al., 2005). Esse teste consiste basicamente na utilização por parte do participante da mão dominante para pressionar a barra de espaço do teclado a sua frente quando qualquer dígito, menos o dígito 3, aparecer na tela do computador. Dessa forma, o participante deverá inibir sua resposta durante a aparição do dígito 3 (ROBERTSON et al., 1997). O número de acertos e tempo do teste será mensurado nas duas aplicações que serão realizadas antes e após a intervenção. Será utilizado o software e-prime para a programação e aplicação do teste.

**Questionários**

Os questionários abaixo descritos serão aplicados antes, durante e depois das intervenções.

**Avaliação multidimensional da consciência interoceptiva - Multidimensional Assessment of interoceptive Awareness – MAIA**

Questionário destinado a mensurar a capacidade de identificar as emoções e sensações corporais interoceptiva (MEHLING, 2012). Os itens são enquadrados em alguns domínios que se referem aos seguintes fatores:

1) Consciência das sensações corporais;

2) Reação emocional e resposta atencional às sensações corporais;

3) Capacidade de regulação emocional;

4) Consciência de Integração mente-corpo;

5) Confiança nas sensações corporais.

O instrumento contém 32 itens e apresenta consistência interna de 0,82. Trabalharemos na validação desse questionário para a população brasileira e utilizaremos a versão traduzida para português (MAIA-VB2).

**FFMQ-** **Questionário das cinco facetas de Mindfulness - Five Facets mindfulness questionnaire (FFMQ)**

Essa escala avalia o nível de *Mindfulness* e é composta por 39 itens, numa escala de Likert de cinco pontos que varia de *(1) nunca ou raramente verdadeiro* a *(5) quase sempre ou sempre verdadeiro*. Nessa escala o conceito de *Mindfulness* está dividido em cinco componentes, são eles:

(1) Observar, que inclui notar ou estar atento a experiências internas e externas, tais como sensações, cognições, emoções, visões, sons e cheiros;

(2) Descrever, que se refere a caracterizar experiências internas através de palavras;

(3) Agir com consciência, que se refere a estar atento às atividades do momento e pode ser contrastado com o comportamento mecânico enquanto a atenção está focada em outra coisa, conhecido como piloto automático;

(4) Não julgamento da experiência interna, que se refere a não tomar uma postura de avaliação e julgamento em relação aos pensamentos e sentimentos;

(5) Não reatividade à experiência interna, que se refere à tendência a permitir que os pensamentos e sentimentos venham e vão sem se deixar afetar ou ser tomado por eles (BAER et al., 2006). Utilizaremos a versão brasileira do questionário (BARROS et al., 2014).

**Escala de mindfulness estado – State Mindfulness Scale (SMS)**

Essa escala visa avaliar níveis de *mindfulness* relacionados a estado de consciência (TANAY; BERNSTEIN, 2013). Foi desenvolvida baseada em escalas de traço de *mindfulness* anteriores a ela e busca a complementação de avaliação de *mindfulness* no momento de sua aplicação. É uma escala que contém 21 itens onde o indíviduo terá de responder às questões com graduação de “nada” a bastante”. Trabalharemos na validação dessa escala para a população brasileira.

**Escala de Estresse Percebido**

A Escala de Estresse Percebido (Perceived Stress Scale - PSS) (COHEN, 1983) é um dos instrumentos mais citados na literatura para estimativa do estresse. A PSS avalia o estresse sob três aspetos: presença de agentes específicos que causam estresse, sintomas físicos e psicológicos do estresse e percepção geral de estresse, independente do seu agente causador (por exemplo, no item 3: “No mês passado, quantas vezes você se sentiu nervoso(a) ou estressado(a)?”). Segundo os autores os demais instrumentos disponíveis na literatura se propõem a estimar o impacto específico de alguns fatores estressantes, o que limita seus resultados dado que eventos relacionados ao estresse podem variar entre indivíduos. Nesse estudo utilizaremos a versão breve dessa escala traduzida e validada para a população brasileira (DIAS et al., 2015)

**Escala de afeto positivo e negativo - Positive and Negative Affect Schedule (PANAS)**

Esse questionário consiste em um conjunto de palavras que descrevem diferentes sentimentos e emoções dispostas em duas escalas de humor com 10 itens cada (WATSON; CLARK; TELLEGEN, 1988). Cada item possui uma pontuação de 1 a 5 pontos (1 = "*muito ligeiramente ou nada*" a 5 = "*extremamente*") para indicar à medida que os entrevistados sentiram os sentimentos e emoções durante as semanas anteriores à entrevista. Utilizaremos a versão validada em língua portuguesa por (GALINHA; PAIS-RIBEIRO, 2005).

**Questionário de avaliação de estado de repouso de Amsterdam ( Amsterdam Resting-state Questionnaire)**

Esse instrumento visa avaliar a relação com os pensamentos em estado de repouso. Desenvolvido por Diaz et al., 2013 e aprimorada em 2014 (ALEXANDER DIAZ et al., 2014; DIAZ et al., 2013), parece de grande valia quando aplicado em participantes submetidos a práticas contemplativas. A versão 2.0 a ser utilizada nesse estudo é composta por 54 itens que deverão ser respondidos em uma escala de 1-5 que varia de “descordo completamente” a “concordo completamente”.

**Inventário de ansiedade Traço- Estado (Strai-Trait Anxiety Inventory- IDATE)**

Este inventário busca avaliar através de duas partes, cada uma contendo 20 afirmações, estado e traço de ansiedade. Foi desenvolvido por Spilberg et al (1970) e traduzido e validado para a população brasileira por Biaggio e Natalício (1979). O participante deve responder em uma escala de 1-4 e o score total de cada escala varia de 20 a 80, sendo valores mais altos indicativos de maiores níveis de ansiedade.

# **Análise Estatística**

Inicialmente serão aplicados testes de normalidade de Kolgomorov-Smirnov para determinar se os dados obedecem a uma distribuição normal. Sendo obedecido este pré-requisito, será aplicado o teste de t de Student (variáveis paramétricas) ou teste U de Mann Whitney (variáveis não paramétricas) para detectar a ocorrência de diferenças significativas intra grupo e ANOVA para verificar diferença entre grupos. O teste de correlação de Spearman também será aplicado para testar a ocorrência de correlações entre as variáveis (depressão, ansiedade, afetividade, estresse e mindfulness traço e estado).

Será realizada uma análise fatorial exploratória e confirmatória para a verificação dos domínios e associação entre os fatores existentes nos questionários. A consistência interna dos fatores será verificada através do Alpha de Crombach e somente os valores de saturação maiores que 0,4 para os autovalores (eigenvalues) serão computados para participação do item no domínio investigado.

Para a análise dos marcadores plasmáticos e comportamentais, serão utilizados testes de correlação e análise multivariada.

Para todos os testes, será considerado o nível de significância (p valor) menor ou igual a 0,05.

**Avaliação dos Riscos e Benefícios:**

**Riscos:**
Durante a coleta de sangue o indivíduo pode sentir vertigem ou uma leve queda de pressão. Qualquer mal-estar será minimizado por um profissional devidamente qualificado e qualquer procedimento necessário a manutenção do quadro de saúde e bem-estar do participante será realizado. Ao ser exposto ao teste de ansiedade social, o participante pode sentir-se nervoso e apresentar sintomas de ansiedade e estresse. No entanto, haverá sempre um profissional junto ao indivíduo provendo qualquer suporte necessário a ele.

**Benefícios:**
Baseado em estudos anteriores, as práticas baseadas em mindfulness para redução do estresse parece modificar a resposta ao estresse via eixo hipotálamo-hipófise-adrenal de maneira a permitir uma melhor adaptação frente a um estímulo estressor. Além disso, sintomas de ansiedade, depressão e estresse percebido são significativamente melhorados após a prática dessas atividades. Adicionalmente, o baixo custo e a fácil execução dessas práticas fundamentam o seu uso na vida diária para a redução do estresse em população clínica ou não clínica. Visando uma possível intervenção com essas características a nível educacional e no sistema público de saúde, os resultados obtidos nesse estudo trarão à luz novas compreensões acerca do seu funcionamento e aplicação.

# **Procedimentos Éticos**

O estudo será realizado de acordo com a "Declaração de Helsinque" (1974), revisada em Tóquio (1975), Veneza (1983), Hong Kong (1989), África do Sul (1996) e Escócia (2000). Resolução CNS 196/196.

Para todos os voluntários, será lido o termo de consentimento, contendo informações sobre justificativa, objetivos, procedimentos, riscos e benefícios do estudo do qual estão sendo convidados a participar. Todos os sujeitos fornecerão consentimento informado por escrito antes da participação.

Os indivíduos que atenderem aos critérios de inclusão serão convidados a participar do ensaio. Todos os pacientes são livres para se retirar a qualquer momento das intervenções do protocolo, sem apresentar razões e sem prejudicar seu tratamento posterior.

A todos os pacientes será garantido o direito de receber informações e esclarecimentos sobre as dúvidas que surgirem no decorrer do protocolo experimental e informações atualizadas sobre o estudo.

A equipe de pesquisa preservará a confidencialidade dos pacientes que participam do estudo. A identidade do paciente não será revelada, bem como todas as informações por ele fornecidas serão mantidas em sigilo.

**Referências**

ALEXANDER DIAZ, B. et al. The ARSQ 2.0 reveals age and personality effects on mind-wandering experiences. **Frontiers in Psychology**, v. 5, n. APR, p. 1–8, 2014.

ALLEVA, E.; SANTUCCI, D. Psychosocial vs. “physical” stress situations in rodents and humans: Role of neurotrophins. **Physiology and Behavior**, v. 73, n. 3, p. 313–320, 2001.

ATANES, A. et al. Validade e confiabilidade da “ Escala de Atencão Plena e Consciência ” ( MAAS ) e “ Questionário das Cinco Facetas de Mindfulness ” ( FFMQ ) entre profissionais da Atenção Primária à Saúde. **Revista Brasileira de Medicina de Família e Comunidade**, p. 2012, 2012.

BAER, R. A. et al. Five Facet Mindfulness Questionnaire. **Assessment**, v. 13, p. 27–45, 2006.

BARROS, V. V. DE et al. Validity evidence of the Brazilian version of the Five Facet Mindfulness. **Psicologia: Teoria e Pesquisa**, v. 30, n. 3, p. 317–327, set. 2014.

BAWA, F. L. M. et al. Does mindfulness improve outcomes in patients with chronic pain? Systematic review and meta-analysis. **The British journal of general practice : the journal of the Royal College of General Practitioners**, v. 65, n. 635, p. e387–e400, 2015.

BERTON, O. et al. Essential role of BDNF in the mesolimbic dopamine pathway in social defeat stress. **Science (New York, N.Y.)**, v. 311, n. 5762, p. 864–8, 2006.

BISHOP, S. R. et al. Mindfulness: A Proposed Operational Definition. **Clinical Psychology: Science and Practice**, v. 11, n. 3, p. 230–241, 11 maio 2004.

BORNEMANN, B. et al. Differential changes in self-reported aspects of interoceptive awareness through 3 months of contemplative training. **Frontiers in Psychology**, v. 6, n. JAN, p. 1–13, 2015.

BOWEN, S. et al. The role of thought suppression in the relationship between mindfulness meditation and alcohol use. **Addictive behaviors**, v. 32, n. 10, p. 2324–8, out. 2007.

BOWEN, S. et al. Relative Efficacy of Mindfulness-Based Relapse Prevention, Standard Relapse Prevention, and Treatment as Usual for Substance Use Disorders. **JAMA Psychiatry**, v. 71, n. 5, p. 547, 1 maio 2014.

BROWN, K. W.; WEINSTEIN, N.; CRESWELL, J. D. Trait mindfulness modulates neuroendocrine and affective responses to social evaluative threat. **Psychoneuroendocrinology**, v. 37, n. 12, p. 2037–2041, 2012.

CHIESA, A.; SERRETTI, A. Mindfulness-based stress reduction for stress management in healthy people: a review and meta-analysis. **The journal of alternative and complementary medicine**, v. 15, n. 5, p. 593–600, 2009.

COHEN, S. **Cohen**, 1983.

COUR, P.; PETERSEN, M. Effects of Mindfulness Meditation on Chronic Pain : A Randomized Controlled Trial. p. 641–652, 2015.

CRESWELL, J. D. et al. Brief mindfulness meditation training alters psychological and neuroendocrine responses to social evaluative stress. **Psychoneuroendocrinology**, v. 44, p. 1–12, 2014.

DAUBENMIER, J. et al. It’s not what you think, it’s how you relate to it: Dispositional mindfulness moderates the relationship between psychological distress and the cortisol awakening response. **Psychoneuroendocrinology**, v. 48, p. 11–18, 2014.

DAVIS, L.; SETH KURZBAN. Mindfulness-Based Treatment for People with Severe Mental Illness: A Literature Review. **American Journal of Psychiatric Rehabilitation**, v. 15, n. 2, p. 202–232, 2012.

DECKRO, G. R. et al. The evaluation of a mind/body intervention to reduce psychological distress and perceived stress in college students. **Journal of American college health : J of ACH**, v. 50, n. 6, p. 281–7, 24 maio 2002.

DELORME, A.; MAKEIG, S. EEGLAB: An open source toolbox for analysis of single-trial EEG dynamics including independent component analysis. **Journal of Neuroscience Methods**, v. 134, n. 1, p. 9–21, 2004.

DIAS, J. C. R. et al. Perceived Stress Scale Applied to College Students: Validation Study. **Psychology, Community & Health**, v. 4, n. 1, p. 1–13, 2015.

DIAZ, B. A. et al. The Amsterdam Resting-State Questionnaire reveals multiple phenotypes of resting-state cognition. **Frontiers in human neuroscience**, v. 7, n. August, p. 446, 2013.

ESCH, T. et al. Mind/body techniques for physiological and psychological stress reduction: stress management via Tai Chi training - a pilot study. **Medical science monitor : international medical journal of experimental and clinical research**, v. 13, n. 11, p. CR488-R497, 2007.

FAN, Y. et al. Short Term Integrative Meditation Improves Resting Alpha Activity and Stroop Performance. **Applied Psychophysiology Biofeedback**, v. 39, n. 3–4, p. 213–217, 2014.

FARB, N. et al. Interoception, contemplative practice, and health. **Frontiers in Psychology**, v. 6, n. June, p. 763, 2015.

FARB, N. A S.; SEGAL, Z. V.; ANDERSON, A. K. Mindfulness meditation training alters cortical representations of interoceptive attention. **Social Cognitive and Affective Neuroscience**, v. 8, n. 1, p. 15–26, 2013.

GALINHA, I. C.; PAIS-RIBEIRO, J. L. Contribuição para o estudo da versão portuguesa da Positive and Negative Affect Schedule (PANAS): II – Estudo psicométrico. **Análise Psicológica**, v. 2, n. XXIII, p. 219–227, 2005.

GODSEY, J. The role of mindfulness based interventions in the treatment of obesity and eating disorders: an integrative review. **Complementary Therapies in Medicine**, v. 21, n. 4, p. 430–439, ago. 2013.

GOLDSTEIN, D. S. Catecholamines and stress. **Endocrine Regulations**, v. 37, n. 2, p. 69–80, 2003.

HASENKAMP, W.; BARSALOU, L. W. Effects of Meditation Experience on Functional Connectivity of Distributed Brain Networks. **Frontiers in Human Neuroscience**, v. 6, n. March, p. 1–14, 2012.

HAUS, E. Chronobiology in the endocrine system. **Advanced Drug Delivery Reviews**, v. 59, n. 9–10, p. 985–1014, 2007.

HOFMANN, S. G. et al. The effect of mindfulness-based therapy on anxiety and depression: A meta-analytic review. **Journal of consulting and clinical psychology**, v. 78, n. 2, p. 169–83, abr. 2010.

HOLZEL, B. K. et al. How Does Mindfulness Meditation Work? Proposing Mechanisms of Action From a Conceptual and Neural Perspective. **Perspectives on Psychological Science**, v. 6, n. 6, p. 537–559, 2011.

HÖLZEL, B. K. et al. Differential engagement of anterior cingulate and adjacent medial frontal cortex in adept meditators and non-meditators. **Neuroscience Letters**, v. 421, n. 1, p. 16–21, 2007.

HORCH, H. W. et al. Destabilization of cortical dendrites and spines by BDNF. **Neuron**, v. 23, n. 2, p. 353–364, 1999.

HUANG, H. PING et al. A meta-analysis of the benefits of mindfulness-based stress reduction (MBSR) on psychological function among breast cancer (BC) survivors. **Breast Cancer**, n. 12, 2015.

JHA, A. P.; KROMPINGER, J.; BAIME, M. J. Mindfulness training modifies subsystems of attention. **Cognitive, affective & behavioral neuroscience**, v. 7, n. 2, p. 109–19, jun. 2007.

KABAT-ZINN, J. **Full Catastrophe Living: Using the Wisdom of Your Body and Mind to Face Stress, Pain, and Illness**. [s.l.] Delta Trade Paperbacks, 1990.

KABAT-ZINN, J.; LIPWORTH, L.; BURNEY, R. The clinical use of mindfulness meditation for the self-regulation of chronic pain. **Journal of behavioral medicine**, v. 8, n. 2, p. 163–90, jun. 1985.

KANG, D. H. et al. The effect of meditation on brain structure: Cortical thickness mapping and diffusion tensor imaging. **Social Cognitive and Affective Neuroscience**, v. 8, n. 1, p. 27–33, 2013.

KILPATRICK, L. A. et al. Impact of mindfulness-based stress reduction training on intrinsic brain connectivity. **NeuroImage**, v. 56, n. 1, p. 290–298, 2011.

KOCH, A.; POLLATOS, O. Cardiac sensitivity in children: Sex differences and its relationship to parameters of emotional processing. **Psychophysiology**, v. 51, n. 9, p. 932–941, 2014.

KOCOVSKI, N. L. et al. Mindfulness and Acceptance-based Group Therapy and traditional Cognitive Behavioral Group Therapy for Social Anxiety Disorder: Mechanisms of Change. **Behaviour Research and Therapy**, v. 70, p. 11–22, 2015.

KOLOTYLOVA, T. et al. Entwicklung des mannheimer multikomponenten-stress-test (MMST). **PPmP Psychotherapie Psychosomatik Medizinische Psychologie**, v. 60, n. 2, p. 64–72, 2010.

KOPIN, I. J. Definitions of stress and sympathetic neuronal responses. **Annals of the New York Academy of Sciences**, v. 771, p. 19–30, 1995.

KURTH, F. et al. Brain Gray Matter Changes Associated with Mindfulness Meditation in Older Adults: An Exploratory Pilot Study using Voxel-based Morphometry. **Neuro : open journal**, v. 1, n. 1, p. 23–26, 2015.

KUYKEN, W. et al. Effectiveness and cost-effectiveness of mindfulness-based cognitive therapy compared with maintenance antidepressant treatment in the prevention of depressive relapse or recurrence (PREVENT): a randomised controlled trial. **The Lancet**, v. 386, n. 9988, p. 63–73, 2015.

LAZAR, S. W. et al. Meditation experience is associated with increased cortical thickness. **Neuroreport**, v. 16, n. 17, p. 1893–7, 28 nov. 2005.

LICINIO, J.; WONG, M.-L. Brain-derived neurotrophic factor (BDNF) in stress and affective disorders. **Molecular Psychiatry**, v. 7, n. 6, p. 519–519, 2002.

LIOTTI, M. et al. Brain responses associated with consciousness of breathlessness (air hunger). **Proceedings of the National Academy of Sciences**, v. 98, n. 4, p. 2035–2040, 2001.

MALINOWSKI, P. Neural mechanisms of attentional control in mindfulness meditation. **Frontiers in Neuroscience**, v. 7, n. 7 FEB, p. 1–11, 2013.

MARCHAND, W. R. Mindfulness-based stress reduction, mindfulness-based cognitive therapy, and Zen meditation for depression, anxiety, pain, and psychological distress. **Journal of Psychiatric Practice**, v. 18, n. 4, p. 233–252, jul. 2012.

MCEWEN, B. S.; WINGFIELD, J. C. What is in a name? Integrating homeostasis, allostasis and stress. **Hormones and behavior**, v. 57, n. 2, p. 105–11, fev. 2010.

MEHLING, W. E. et al. The Multidimensional Assessment of Interoceptive Awareness (MAIA). **PLoS ONE**, v. 7, n. 11, 2012.

MEHLING, W. E. Multidimensional Assessment of Interoceptive Awareness. v. 1, n. 415, 2012.

MURAKAMI, S. et al. Chronic stress, as well as acute stress, reduces BDNF mRNA expression in the rat hippocampus but less robustly. **Neuroscience Research**, v. 53, n. 2, p. 129–139, 2005.

O’LEARY, K.; O’NEILL, S.; DOCKRAY, S. A systematic review of the effects of mindfulness interventions on cortisol. **Journal of health psychology**, n. March 2016, p. 1359105315569095-, 2015.

O’REILLY, G. et al. Minfulness-Based Interventions for Obesity-Related Eating Behaviors: A Literature Review. **Obes Rev**, v. 15, n. 6, p. 453–461, 2014.

REINHARDT, T. et al. Salivary cortisol, heart rate, electrodermal activity and subjective stress responses to the Mannheim Multicomponent Stress Test (MMST). **Psychiatry Research**, v. 198, n. 1, p. 106–111, 2012.

RICCIO, C. A. et al. The continuous performance test: A window on the neural substrates for attention? **Archives of Clinical Neuropsychology**, v. 17, n. 3, p. 235–272, 2002.

ROBERTSON, I. H. et al. “Oops!”: Performance correlates of everyday attentional failures in traumatic brain injured and normal subjects. **Neuropsychologia**, v. 35, n. 6, p. 747–758, 1997.

SANDERS, A. F. Towards a Model of Stress and Human. **Acta Psychologica**, v. 53, p. 61–97, 1983.

SAPOLSKY, R. M.; ROMERO, L. M.; MUNCK, A. U. How Do Glucocorticoids Influence Stress Responses ? Preparative Actions *. **Endocrine Reviews**, v. 21, n. April, p. 55–89, 2000.

SCHANDRY, R. Heart Beat Perception and Emotional Experience. **Psychophysiology**, v. 18, n. 4, p. 483–488, 1981.

SELMAOUI, B.; TOUITOU, Y. Reproducibility of the circadian rhythms of serum cortisol and melatonin in healthy subjects: A study of three different 24-h cycles over six weeks. **Life Sciences**, v. 73, n. 26, p. 3339–3349, 2003.

STRATAKIS, C. A.; CHROUSOS, G. P. Neuroendocrinology and pathophysiology of the stress system. **Annals of the New York Academy of Sciences**, v. 771, p. 1–18, 1995.

TAKAHASHI, M. et al. Abnormal expression of brain-derived neurotrophic factor and its receptor in the corticolimbic system of schizophrenic patients. **Molecular psychiatry**, v. 5, n. 3, p. 293–300, 2000.

TANAY, G.; BERNSTEIN, A. State Mindfulness Scale (SMS): development and initial validation. **Psychological assessment**, v. 25, n. 4, p. 1286–99, 2013.

TANG, Y.-Y. et al. Short-term meditation training improves attention and self-regulation. **Proceedings of the National Academy of Sciences of the United States of America**, v. 104, n. 43, p. 17152–17156, 2007.

TANG, Y.-Y.; HÖLZEL, B. K.; POSNER, M. I. Traits and states in mindfulness meditation. **Nature Reviews Neuroscience**, v. 17, n. DECEMBER, p. 59–59, 2015a.

TANG, Y.-Y.; HÖLZEL, B. K.; POSNER, M. I. The neuroscience of mindfulness meditation. **Nature Reviews Neuroscience**, v. 16, n. 4, p. 1–13, 2015b.

TSIGOS, C.; CHROUSOS, G. P. Hypothalamic-pituitary-adrenal axis, neuroendocrine factors and stress. **Journal of Psychosomatic Research**, v. 53, n. 4, p. 865–871, 2002.

UEBELACKER, L. A.; BROUGHTON, M. K. Yoga for Depression and Anxiety: A Review of Published Research and Implications for Healthcare Providers. **Rhode Island medical journal (2013)**, v. 99, n. 3, p. 20–2, jan. 2016.

ULRICH-LAI, Y. M.; HERMAN, J. P. Neural regulation of endocrine and autonomic stress responses. **Nature reviews. Neuroscience**, v. 10, n. 6, p. 397–409, 2009.

VALDEZ, P. et al. Circadian rhythms in components of attention. **Biological Rhythm Research**, v. 36, n. 1–2, p. 57–65, 2005.

WANG, F. et al. The effects of tai chi on depression, anxiety, and psychological well-being: a systematic review and meta-analysis. **International journal of behavioral medicine**, v. 21, n. 4, p. 605–17, ago. 2014.

WATSON, D.; CLARK, L. A; TELLEGEN, A. Development and validation of brief measures of positive and negative affect: the PANAS scales. **Journal of personality and social psychology**, v. 54, n. 6, p. 1063–1070, 1988.

WÜRTZEN, H. et al. Effect of mindfulness-based stress reduction on somatic symptoms, distress, mindfulness and spiritual wellbeing in women with breast cancer: Results of a randomized controlled trial. **Acta Oncologica**, v. 54, n. August 2014, p. 1–8, 2015.

ZHOU, R.; LIU, L. Eight-Week Mindfulness Training Enhances Left Frontal EEG Asymmetry During Emotional Challenge: a Randomized Controlled Trial. **Mindfulness**, p. 1–9, 2016.

ZYLOWSKA DEBORAH ACKERMAN MAY H YANG JULIE L FUTRELL NANCY L HORTON T SIGI HALE, L. L.; PATAKI, C.; SMALLEY, S. L. Mindfulness Meditation Training in Adults and Adolescents With ADHD A Feasibility Study. **Journal of Attention Disorders**, v. 11, n. 6, p. 737–746, 2008.

**Transcrição do áudio- Grupo Mindfulness (30 minutos)**

Obrigada por fazer parte da nossa pesquisa

Adquira uma posição confortável na qual você consiga passar os próximos minutos.

Se houver algum pensamento, algum julgamento, apenas se de conta disso e gentilmente retorne a sua atenção até a sua respiração.

De olhos fechados, convide-se neste instante a trazer a sua atenção até o seu corpo

Perceba todas as sensações que o seu corpo te oferece

Desde a temperatura do ambiente, o contato da sua roupa com a sua pele, o contato do seu corpo com a superfície na qual você está.

Talvez você possa passar sua atenção por todas as partes do seu corpo, desde os seus pés, percebendo também o seu quadril, o contato do seu quadril com a superfície na qual você está

Percebendo também o seu abdome e a mudança de forma que ocorre nessa região a medida em que você inspira e expira.

Perceba também o seu tórax, note os movimentos que ele faz junto com a sua respiração.

Perceba o seu corpo respirar nesse instante. Lembre-se que não é necessário alterar ou controlar a sua respiração. Apenas siga o seu próprio ritmo.

Se em algum momento você perceber que se engajou em pensamentos, gentilmente retorne a sua atenção para o seu corpo.

Perceba os movimentos que o seu corpo faz a medida em que você inspira e expira.

Volte sua atenção para o seu corpo. Perceba se existe algum desconforto neste instante. Se existir, direcione a sua respiração para essa região de desconforto e libera toda e qualquer tensão junto com a sua respiração.

Perceba como seu corpo reage ao ar que entra e sai do seu corpo.

Apenas repouse a sua atenção sobre esse movimento, apenas repouse a sua consciência sobre a sua respiração.

Se você perceber algum pensamento, algum julgamento, com gentileza e curiosidade, retorne a sua atenção para a sua respiração. Perceba todas as sensações que a sua respiração te oferece.

Convide-se agora a levar sua atenção até a região entre o seu lábio superior e o seu nariz. Talvez você possa perceber o fluxo de ar que entra e sai do seu corpo.

Se você perceber muitos pensamentos, apenas deixe-os passar e retorna sua atenção para a sua respiração.

Talvez você possa perceber se existe alguma diferença de temperatura entre a sua inspiração e sua expiração.

Perceba também se existe alguma diferença entre a sua narina direita e a sua narina esquerda. Note se o fluxo de ar entre essas duas regiões é diferente. Apenas observe.

Perceba a temperatura e a quantidade de ar que entra e sai do seu corpo.

Volte a sua atenção para a sua respiração.

Volte a sua atenção para o seu corpo. Perceba os movimentos que o seu corpo faz a medida em que você inspira e expira.

Lembre-se que não é preciso se julgar ou se culpar caso você se perca em meio a pensamentos.

Se isso acontecer, se de conta disso e retorne gentilmente a sua atenção para a sua respiração, deixando todo e qualquer pensamento passar.

Volte a sua atenção para o seu corpo. Perceba como você

se sente neste instante.

Note se existe algum desconforto no seu corpo, e se existir, direcione a sua respiração para essa região e libere este desconforto junto com a sua respiração.

Recepcione todas as sensações que o seu corpo te oferece.

Perceba os movimentos que o seu corpo faz a medida em que você inspira e expira.

Perceba a temperatura do ambiente.

Perceba o contato do seu corpo com a superfície na qual você está.

Perceba como se sente aqui, agora.
